# Small peptide-based GLP-1R ligands: an approach to reduce the kidney uptake of radiolabeled GLP-1R-targeting agents?

**DOI:** 10.1186/s41181-021-00136-x

**Published:** 2021-08-25

**Authors:** Veronika Barbara Felber, Hans-Jürgen Wester

**Affiliations:** Chair of Pharmaceutical Radiochemistry, Technical University of Munich, Walther-Meißner-Str. 3, 85748 Garching, Germany

**Keywords:** GLP-1R, Small peptides, Insulinoma, Diagnosis, Peptide receptor radionuclide therapy, Kidneys

## Abstract

**Aim:**

Elevated kidney uptake in insulinoma patients remains a major limitation of radiometallated exendin-derived ligands of the glucagon-like peptide 1 receptor (GLP-1R). Based on the previously published potent GLP-1R-activating undecapeptide **1**, short-chained GLP-1R ligands were developed to investigate whether kidney uptake can be reduced by means of direct ^18^F-labeling (nuclide-based accelerated renal excretion) or the reduction of the overall ligand charge (ligand-based reduced kidney uptake).

**Materials & methods:**

GLP-1R ligands were prepared according to optimized standard protocols via solid-phase peptide synthesis (SPPS) or, when not practicable, via fragment coupling in solution. Synthesis of (2‘-Et, 4‘-OMe)4, 4’-L-biphenylalanine ((2′-Et, 4′-OMe)BIP), required for the preparation of **1**, was accomplished by Suzuki-Miyaura cross-coupling. In vitro experiments were performed using stably transfected GLP-1R^+^ HEK293-hGLP-1R cells.

**Results:**

In contrast to the three reference ligands glucagon-like peptide 1 (GLP-1, IC_50_ = 23.2 ± 12.2 nM), [Nle^14^, Tyr(3-I)^40^]exendin-4 (IC_50_ = 7.63 ± 2.78 nM) and [Nle^14^, Tyr^40^]exendin-4 (IC_50_ = 9.87 ± 1.82 nM), the investigated GLP-1R-targeting small peptides (9–15 amino acids), including lead peptide **1**, exhibited only medium to low affinities (IC_50_ > 189 nM). Only SiFA-tagged undecapeptide **5** (IC_50_ = 189 ± 35 nM) revealed a higher affinity than **1** (IC_50_ = 669 ± 242 nM).

**Conclusion:**

The investigated small peptides, including lead peptide **1***,* could not compete with favorable in vitro characteristics of glucagon-like peptide 1 (GLP-1), [Nle^14^, Tyr(3-I)^40^]exendin-4 and [Nle^14^, Tyr^40^]exendin-4. The auspicious EC_50_ values of **1** provided by the literature could not be transferred to competitive binding experiments. Therefore, the use of **1** as a basic scaffold for the design of further GLP-1R-targeting radioligands cannot be recommended. Further investigations might include the scaffold of **5**, although substantial optimizations concerning affinity and lipophilicity would be required. In sum, GLP-1R-targeting radioligands with reduced kidney uptake could not be obtained in this work, which emphasizes the need for further ligands addressing this particular issue.

**Supplementary Information:**

The online version contains supplementary material available at 10.1186/s41181-021-00136-x.

## Introduction

For imaging of insulinoma lesions, a variety of exendin-4-based ligands have been or are currently investigated in clinical trials for targeting the glucagon-like peptide 1 receptor (GLP-1R) (Jansen et al., 2019; https://clinicaltrials.gov/ct2/results?cond=Insulinoma&term=glp-1R&cntry=&state=&city=&dist=&Search=Search, 2020). Labeled with ^18^F, ^125^I, ^68^Ga, ^99m^Tc, ^89^Zr or ^111^In, these compounds show high potential for visualization of primary tumors and metastases. Whereas radiohalogenated compounds exhibit low kidney uptake and/or fast renal excretion (Lappchen et al., 2017; Kiesewetter et al., 2012a), elevated accumulation and retention in the kidneys (> 140% ID/g, 1–4 h p.i.) is always observable when residualizing radiometals are used for exendin-4 derivatization (Jansen et al., 2019; Kiesewetter et al., 2012b; Xu et al., 2015). This non-target tissue uptake not only impairs the detection of insulinomas but could also affect the localization of other GLP-1R positive tumors like pheochromocytomas and gastrinomas, due to the close local proximity of the kidneys to the respective target organs (pancreas (insulinoma), adrenal medulla (pheochromocytoma), duodenum, pancreas and periduodenal lymph nodes (gastrinoma)) (Luo et al., 2015; Sbardella & Grossman, 2020; Donow et al., 1991). Thereby, the instrinsically lower resolution of single-photon emission computed tomography (SPECT) revealed an inferior rate of detection compared to positron emission tomography (PET) (Velikyan & Eriksson, 2020). Moreover, the radiation burden of the kidneys might rapidly become unacceptably high with long-lived radioisotopes, especially in the context of peptide receptor radionuclide therapy (PRRT) approaches (Jansen et al., 2019).

A conventional method used for lowering the kidney uptake of radiotracers makes use of pre-administration of amino acids (Rolleman et al., 2003). Gotthardt et al. applied this method for [^111^In]In-DTPA-exendin-4 in rats and revealed that both gelofusine (18.7% decrease) and poly-L-glutamic acid (29.4% decrease) as well as the combination of both (47.9% decrease) had a significant impact on kidney uptake, whereas the administration of L-lysine did not show any effect (Gotthardt et al., 2007). Besides, coinfusion of albumin-derived peptide fragments reduced the kidney uptake of [^111^In]In-DTPA-exendin-3 by 26% in rats (Vegt et al., 2010).

In order to circumvent any co−/pre-administration steps, modifications of the exendin-4 scaffold itself were pursued to improve the tumor-to-kidney ratio. As a possible strategy, radiotracers with kidney-cleavable linkers have been introduced (Yim et al., 2013; Jodal et al., 2015), and resulted in a 50% reduction of radiation dose in mice, although a general proof-of-concept in humans is still pending (Zhang et al., 2019). For diagnostic purposes, studies with ^18^F-labeled exendin-4 revealed high tracer uptake in INS-1 tumor cells and xenograft models and a rapid clearance from the kidneys compared to radiometallated/[^18^F]AlF analogs (Kiesewetter et al., 2012a; Mikkola et al., 2016; Gao et al., 2011; Wu et al., 2013). Although these results suggested high potential of ^18^F-labeled exendin-4 derivatives, none of the investigated compounds were transferred into clinical application (Jansen et al., 2019; https://clinicaltrials.gov/ct2/results?cond=Insulinoma&term=glp-1R&cntry=&state=&city=&dist=&Search=Search, 2020). Occasionally, difficulties in separating the precursor from the respective radiolabeled agent resulted in low molar activities (A_m_) (Wu et al., 2013; Yue et al., 2014). As a consequence, optimal tumor uptake could not be attained, since a low peptide dose (~ 1 μg) and hence, a high molar activity (~ 200 GBq/μmol (Boerman & Gotthardt, 2012)) is mandatory. Moreover, dependent on the labeling methodology, a relatively high nonspecific accumulation in liver and intestines occurred in preclinical studies (Kiesewetter et al., 2012a; Gao et al., 2011). Direct ^18^F-labeling via a SiFA-modified precursor provided only low ^18^F-incorporation (6%), low radiochemical yields (RCY, 1.0–1.5%) and a slow blood clearance (3.3 ± 0.8%ID/g, 2 h p.i.) (Dialer et al., 2018). Moreover, PRRT of GLP-1R-overexpressing tumors has not been envisaged yet. Potential nephrotoxicity disallows the use of radiometallated α- or β^−^-emitting agents and for ^131^I-labeled derivatives, an ingenious treatment protocol with Irenat® would be indispensable to avoid severe ^131^I-induced damage of the thyroid (Lappchen et al., 2017). In consequence, the use of the exendin-3/4 scaffold currently allows for a very limited number of peptide-radionuclide conjugates that can be only applied for insulinoma imaging in patients.

Since a low amount of GLP-1R expression was detected in the kidneys and high activity accumulation by this organ could not be specifically blocked by an excess of unlabeled analog, a non-saturable, GLP-1R independent mechanism was presumed for tubular reabsorption (Gotthardt et al., 2006; Brom et al., 2010; Brom et al., 2012). Accordingly, further studies revealed that the megalin transporter system of the renal proximal tubules is crucial for uptake and retention of ^111^In-labeled exendin-4 and potential metabolites (Gotthardt et al., 2007; Jodal et al., 2015; Vegt et al., 2011). Kidney extracts of mice that received [^18^F]AlF-NOTA-exendin-4 showed a single very polar radioactive metabolite at 1 h p.i. and no detectable parent peptide. The identity of this metabolite could not be determined, but the general observation that exendin-4 peptides with *C*-terminal radiometal chelates exhibit very high uptake suggests that some metabolite from the *C*-terminal end might be responsible for the slow egress from the kidneys (Kiesewetter et al., 2012b). By comparing the renal accumulation of the ^111^In-labeled G protein-coupled receptor (GPCR) ligands octreotide, minigastrin, bombesin and exendin-4, Gotthardt et al. observed that the number of charged amino acids in these peptides correlates with their kidney uptake. Therefore, beside radiometal chelate-induced renal retention, the high number of charged amino acids of exendin-4-based radioligands is supposed to play a critical role for tubular reabsorbtion, although the exact mechanism still remains unknown (Gotthardt et al., 2007).

Based on the aforementioned study, we hypothesized that downsized GLP-1R-binding structures might have the advantage to exhibit a reduced kidney uptake and might provide accelerated urinary excretion, due to their inherent reduced number of charged amino acids. However, at present, GLP-1R-targeting radioligands, that structurally clearly differ from exendin-4 are not available. Although low-molecular-weight organic molecules were developed for addressing the GLP-1R (orally available antidiabetics) (Donnelly, 2012; Graaf et al., 2016), none of them was able to show favorable properties comparable or even superior to exendin-4/GLP-1-based polypeptide ligands (Graaf et al., 2016; Willard et al., 2012; Knudsen et al., 2007). As a result, these structures were not considered for further use as GLP-1R-targeted radiopharmaceuticals.

**Fig. 1:**
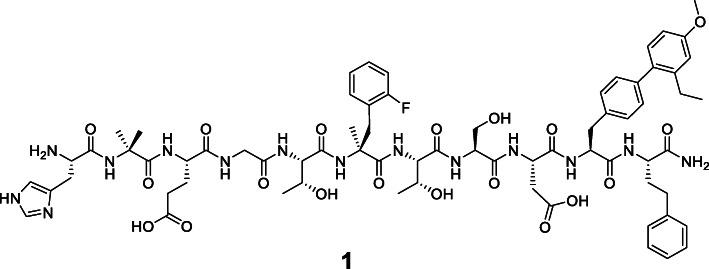
Sequence of lead peptide **1**, with His^1^-Aib^2^-Glu^3^-Gly^4^-Thr^5^-(α-Me)Phe(2-F)^6^-Thr^7^-Ser^8^-Asp^9^-(2′-Et, 4′-OMe)BIP^10^-homoPhe^11^-NH_2_ (Haque et al., 2010a). All amino acids are in L-configuration and given in the three-letter code. Et, ethyl; Me, methyl; BIP, 4, 4′-L-biphenylalanine.

By contrast, short-chained undecapeptides developed in 2009 by Mapelli et al. were able to induce 3′, 5′-cyclic adenosine monophosphate (cAMP) production in chinese hamster ovary (CHO) cells, stably overexpressing the human GLP-1 receptor with similar EC_50_ values (87 pM) like GLP-1 (34 pM; in this study GLP-1 refers to GLP-1(7-36)amide unless otherwise stated) (Mapelli et al., 2009). Indeed, these high potencies were not plausible at first sight, since downsizing of GLP-1R ligands was assumed to lead to non-binding and hence, non-signaling agonists (Parker et al., 1998; Hjorth et al., 1994). However, the bulky hydrophobic residues at the *C*-terminus (position 10 and 11) of these GLP-1 mimetics, obviously compensate for the 21-residue GLP-1(16-36)amide fragment to a certain extent. 2-Aminoisobutyric acid (Aib) instead of L-alanine was introduced at the second position to confer dipeptidyl peptidase IV (DPP IV) resistance. For stabilization of an α-helical structure presumed to be crucial, also for binding of the endogenous ligand GLP-1 (Underwood et al., 2010), L-phenylalanine at position 6 was substituted with L-α-methyl-(2-fluoro)-phenylalanine. Additional screening experiments for alternative residues at positions 10 and 11, revealed lead peptide **1** which was even more potent (EC_50_ = 31 pM) bearing a simple L-homophenylalanine residue at position 11 and a more bulky (2‘-Et, 4‘-OMe)4, 4’-L-biphenylalanine at the penultimate position (Fig. 1) (Haque et al., 2010a; Haque et al., 2010b). Unfortunately, affinity data were not presented in these studies.

Follow-up studies, based on these undecapeptides mainly focused on GLP-1 peptidomimetics with cyclic constraints or cyclic α-conotoxin-GLP-1 chimeras (Hoang et al., 2015; Swedberg et al., 2015; Swedberg et al., 2016). These modifications led to a clear activity decline in cAMP signaling (EC_50_) in comparison to the respective non-cyclized 11-mer peptides.

Although none of these undecapeptides were converted into an imaging agent for insulinomas so far, they might potentially serve as a basis for GLP-1R radioligand with an improved pharmacokinetic profile. More precisely, reabsorption by megalin on the tubular cells and secretion of radioactive degradation products into the blood might be decreased for small peptides (< 30 amino acids (Liu & Edwards, 1999)) derived from **1**, as this active process is possibly triggered to a lesser extent by molecules structurally altered to exendin-3/4 and exhibiting fewer charged amino acid residues (Gotthardt et al., 2007; Jodal et al., 2015; Vegt et al., 2011; Tojo & Kinugasa, 2012). Accelerated clearance from the blood and kidneys would result in earlier adequate tumor-to-background and tumor-to-kidney ratios, particularly advantageous for diagnostic and therapeutic applications, which might be also combined in a theranostic approach (Weineisen et al., 2015), provided that agonist-induced side effects (hypoglycemia, nausea, vomiting) are still tolerable.

In addition, cumbersome labeling procedures as currently required for radiofluorination might become dispensable if the respective ligands would allow for derivatization with a silicon-based fluoride acceptor moiety. Improved labeling conditions in combination with the ‘Munich method’ (Wessmann et al., 2012) for ^18^F-drying might enable direct radiofluorination with higher RCYs, exploiting the favorable imaging characteristics of ^18^F (low β^+^ energy of max. 0.635 MeV, β^+^ decay ratio of 97%, half-life 109.8 min) and the possibility of generating GLP-1R-targeting radiohybrid ligands (Jacobson et al., 2015; Wurzer et al., 2020).

Thus, the aim of this study was to investigate whether undecapeptide **1** (Fig. 1) might serve as an alternative lead structure for GLP-1R targeting, based on its favorable GLP-1R-activating properties (EC_50_ (**1**) = 31 pM vs. EC_50_ (GLP-1) = 34 pM (Mapelli et al., 2009; Haque et al., 2010a)).

Derivatives of **1,** suitable for direct radiolabeling by isotopic exchange on a SiFA-bearing prosthetic group, that could also be valuable precursors for future studies on the insertion of other modifications (e.g. attachment of a chelator), were planned to be preselected by affinity determinations. As a result, the main objective of this study was to generate precursor molecules with low nanomolar IC_50_ values that preferably lie in the range of endogenous GLP-1.

## Materials & methods

For detailed information on all methods for synthesis and analysis as well as on the used instruments, see the supporting information (available on https://ejnmmipharmchem.springeropen.com).

### Chemical synthesis

The GLP-1R ligands were prepared according to optimized standard protocols via solid-phase peptide synthesis (SPPS) or, when not practicable, via fragment coupling in solution. Final purification of the compounds was achieved by RP-HPLC. For a detailed description of the synthesis of derivatives **1–14** see the ‘METHODS’ section in the supporting information (SI). Schematic illustrations of derivatives **1, 2** as well as **4** to **14** are depicted in this manuscript, whereas the structural formula of the radioiodinated reference [Nle^14^, [^125^I]Tyr (3-I)^40^]exendin-4 ([^125^I]Tyr(3-I)^40^–**3**) is given in the SI.

### Radiolabeling

^*125*^*I-labeling.* The radioiodinated reference ligand [Nle^14^, [^125^I]Tyr(3-I)^40^]exendin-4 ([^125^I]Tyr(3-I)^40^–**3**) was prepared via the Iodogen method (Salacinski et al., 1981), with some minor modifications. The precursor [Nle^14^, Tyr^40^]exendin-4 (150 μg, 30.6 nmol, 122 eq.) was dissolved in 20 μL DMSO and 280 μL TRIS buffer (25 mM TRIS HCl, 0.04 M NaCl, pH = 7.5). The solution was transferred to a vial, which has been precoated with 15.0 μg Iodogen (34.7 nmol, 139 eq.). Afterwards, 15 ± 5 MBq [^125^I] NaI (74 MBq/nmol, 3.7 GBq/mL in 40 mM NaOH, Hartmann Analytic, Braunschweig, Germany) (5.00 μL, 250 pmol, 1.00 eq.) were added, the reaction solution was incubated for 15 min at r.t. and 150 μL thereof were purified by radio-RP-HPLC (0% B (2 min) ➔ 0–37.5% B (3 min) ➔ 37.5% B (35 min) ➔ 38% B (10 min), Method A*, 1 mL/min) to afford 3.15 MBq (48.8% RCY, > 99% RCP) of product [^125^I]Tyr(3-I)^40^–**3**. The radioligand was stored in the HPLC solvent (stock solution) at − 80 °C until further use. The radioligand stock solution was used to a maximum of 14 days to ensure a RCP of > 92% (Table 1 in the Additional file 1) and replaced by freshly prepared radioligand afterwards. Prior to the in vitro experiments, the radioligand was dissolved in HBSS (with or w/o 1% BSA) to achieve a final concentration of 0.41 nM in the respective assays. *Method A: solvent A = water + 0.1% TFA, solvent B = acetonitrile + 2% water + 0.1% TFA.

### In vitro experiments

#### Cell culture

HEK293-hGLP-1R cells (HEK293 cells stably transfected with the human GLP-1 receptor) (Gromada et al., 1995) were kindly provided by Prof. Dr. Timothy Kieffer (University of British Columbia, Vancouver, Canada). Cells were cultivated in high glucose DMEM (25.0 mM D-glucose, 3.97 mM GlutaMAX; REF-number: 61965–026; Fisher Scientific GmbH, Schwerte, Germany) supplemented with 10% fetal bovine serum and 1 mM sodium pyruvate. Medium for stably transfected HEK293-hGLP-1R cells was constantly supplemented with 1 mg/mL Geneticin (G-418 Biochrom, Merck KgaA, Darmstadt, Germany) as selection antibiotic. The cell line was kept at 37 °C in a humidified 5% CO_2_ atmosphere. One day (24 ± 2 h) prior to all in vitro experiments, the cultivated HEK293-h-GLP-1R cells were harvested using a mixture of trypsin/ethylenediaminetetraacetic acid (0.05%/0.02%) in phosphate-buffered saline (PBS) (Gibco, Germany) and centrifuged at 1300 rpm (ca. 190×g) for 3 min at room temperature (Heraeus Megafuge 16, Thermo Fisher, Germany). After centrifugation, the supernatant was disposed and the cell pellet was resuspended in culture medium. Cells were counted with a Neubauer hemocytometer (Paul Marienfeld GmbH & Co. KG, Lauda-Königshofen, Germany) and seeded in 24-well plates. IC_50_ values were determined by transferring 1.50 × 10^5^ cells/mL per well into 24-well plates.

#### Affinity determinations (IC_50_)

Detailed information on affinity experiments is provided in the SI. In brief, competitive binding studies were determined on HEK293-hGLP-1R cells (1.50 × 10^5^ cells in 1 mL/well) after incubation at 4 °C for 2 hours, using [^125^I]Tyr(3-I)^40^–**3** (0.41 nM/well) as reference radioligand (*n* = 3). IC_50_ values of GLP-1 were determined in parallel on the same day to ensure assay validity. Only IC_50_ values of the new compounds, for which the IC_50_ of the corresponding GLP-1 control experiment lied in a range of 23.2 ± 12.2 nM (*n* = 11), were considered.

## Results

### Synthesis

**Scheme 1:**
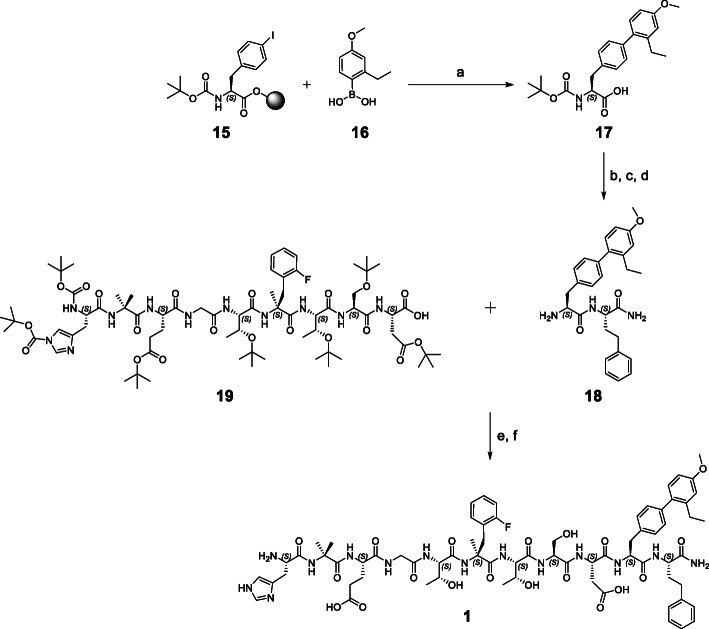
Synthetic route for the preparation of lead peptide **1**. **a** Pd(PPh_3_)_4_, K_3_PO_4_ [DMA], 80 °C, 22 h, argon; **b** H-L-homoPhe-RACM, PyBOP, HOAt, DIPEA [DMF/DCM], r.t., 24 h; **c** TFA/TIPS/H_2_O; **d** tetraalkylammonium carbonate [THF], r.t., 2 h; **e** HOAt, DIC [DMF/DCM], r.t., 19 h; **f** TFA/TIPS/H_2_O. Detailed synthesis procedures are given in the supporting information

Small peptide GLP-1R ligands **2** to **14** (except **3** and Tyr(3-I)^40^–**3**) were synthesized completely on Rink amide ChemMatrix® (RACM) resin. Synthesis of lead peptide **1** had to be accomplished via fragment coupling in solution (Scheme 1). Despite several attempts to realize on-resin synthesis as described by Mapelli et al. (Mapelli et al., 2009), **1** could not be obtained by a linear reaction protocol.

**Fig. 2:**
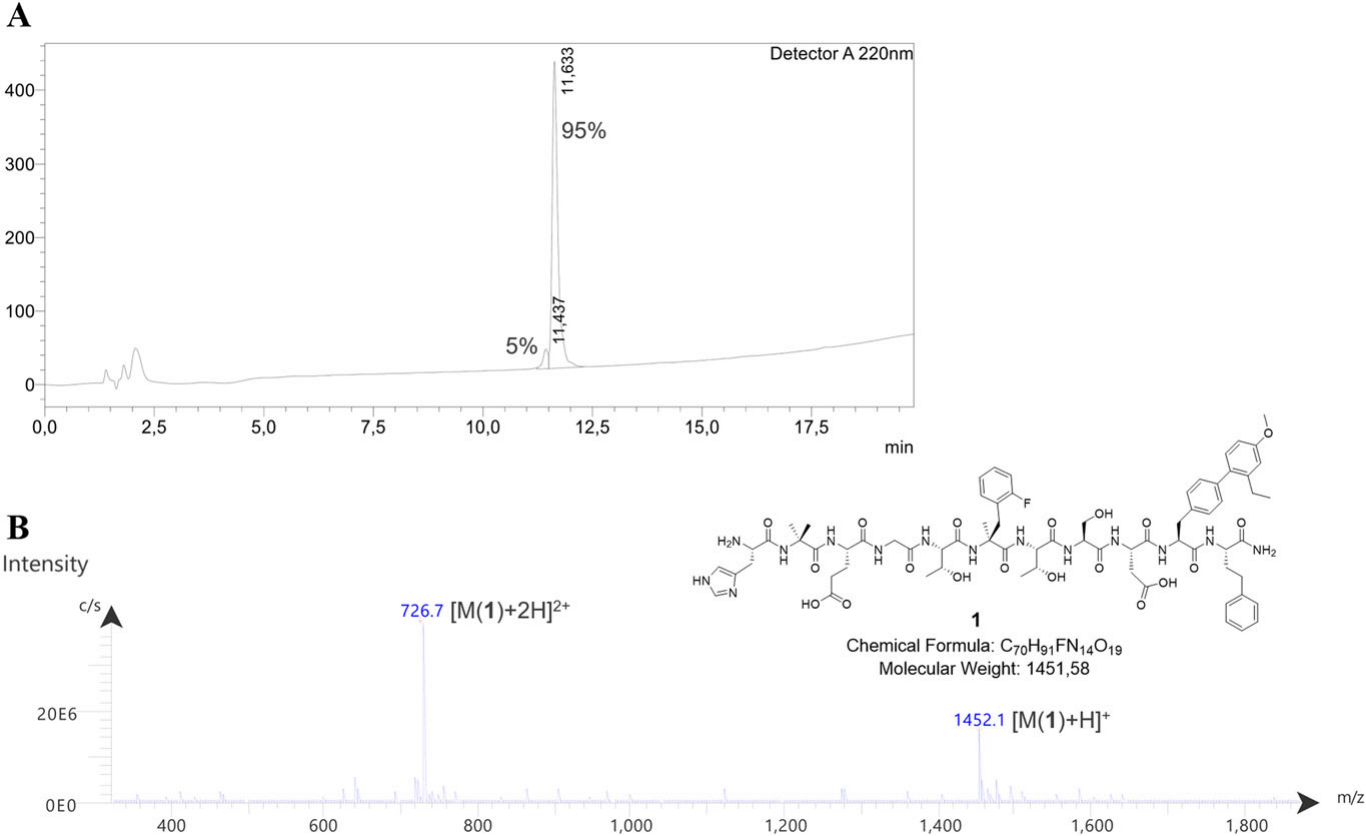
**A** HPLC chromatogram of purified lead peptide **1** (20–80% B in 15 min, Method A*, 1 mL/min, t_R_ = 11.6 min), which was obtained in 95% chemical purity. **B** Corresponding mass spectrum of the HPLC-fraction collected at 11.6 min. *Method A: solvent A = water + 0.1% TFA, solvent B = acetonitrile + 2% water + 0.1% TFA

On-resin Suzuki-Miyaura cross-coupling using compounds **15** and **16** provided the (2′-Et, 4′-OMe)BIP fragment **17** still functionalized with a cross-coupling resistant Boc-protective group at the *N-*terminus. Cleavage of **17** from the resin by HFIP/DCM (1/4) was not mandatory, since a major part was already cleaved from the 2-CT resin by the cross-coupling conditions. Boc-protected **17** was coupled to RACM resin-bound H-L-homoPhe and subsequent cleavage with TFA/TIPS/H_2_O (95/2.5/2.5) revealed dipeptide **18**. Fragment coupling of purified **18** and **19** provided lead peptide 1*2TFA as colorless powder (2.05 mg, 16.1% referred to **18**) after removal of all acid-labile protective groups and RP-HPLC purification (Fig. 2). This strategy involved three RP-HPLC purifications steps in total, after synthesis of **17**, **18** and **1**.

### In vitro characterization

For direct comparison, IC_50_ data of all new derived agents as well as of reference compounds **1**, **2**, **3** and Tyr(3-I)^40^–**3** are displayed in Table 1. Due to different in vitro displacement behaviors initially observed for **4** and **5**, SiFA-tagged peptides were investigated in assays supplemented with (w/) and without (w/o) BSA to determine optimum binding conditions.

**Table 1:**
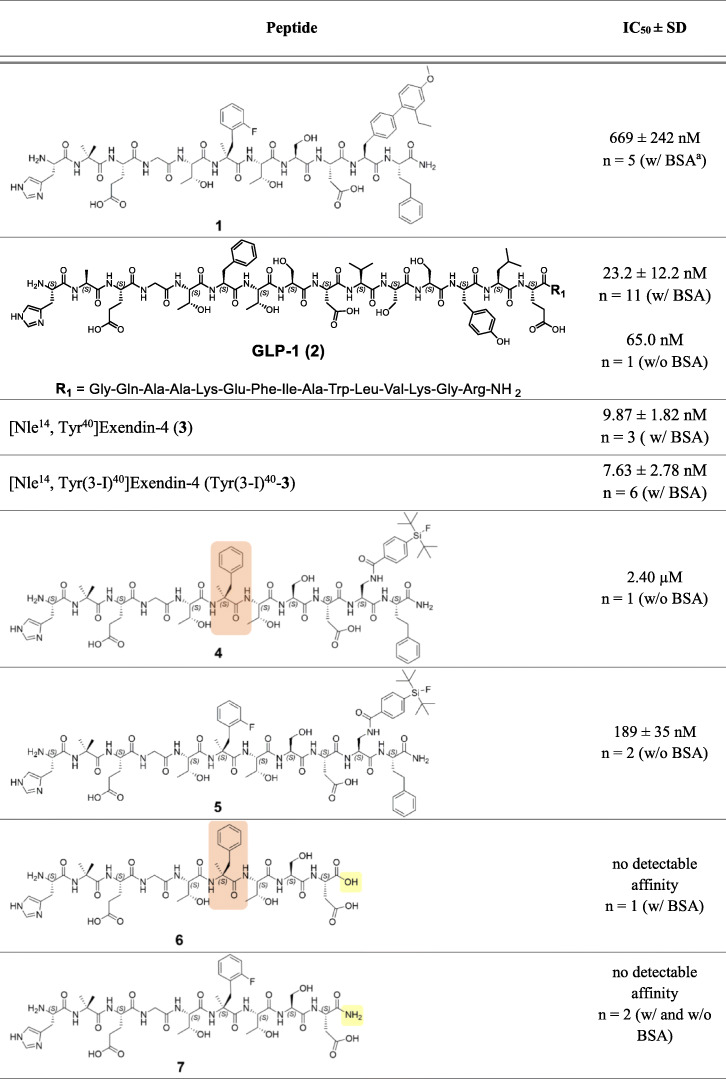

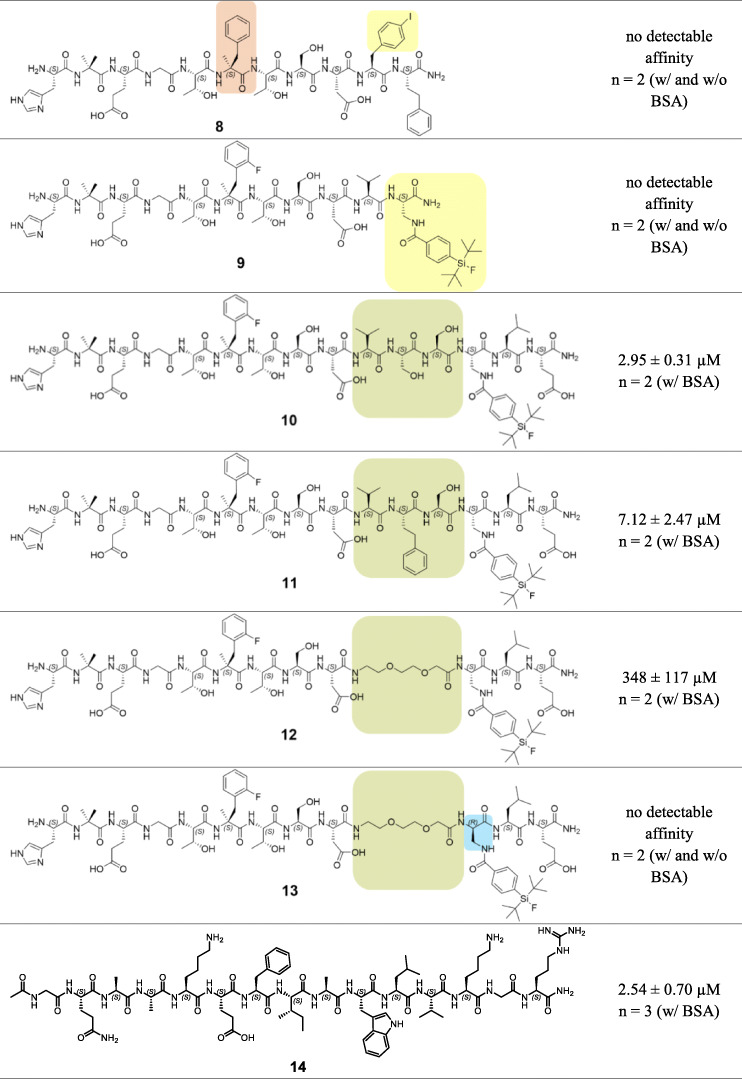
IC_50_ data of SiFA-tagged undecapeptides (**4** and **5**), peptides without C-terminal bulky substituents (**6**, **7** and **8**), SiFA-tagged chimeras of **1** and GLP-1 (**9** to **13**) as well as the *C*-terminal pentadecapeptide of GLP-1 (**14**) developed for targeting the GLP-1 receptor. IC_50_ values of reference ligands **1**, GLP-1 (**2**), [Nle^14^, Tyr^40^]exendin-4 (**3**) and [Nle^14^, Tyr(3-I)^40^]exendin-4 (Tyr(3-I)^40^–**3**) are depicted above

*SD* standard deviation; assay conditions are indicated in brackets after the number of experiments

^a^‘w/ BSA’ indicates supplementation with 1% BSA, whereas ‘w/o BSA’ indicates no supplementation with BSA

## Discussion

### SiFA-tagged undecapeptides

Based on the assumption, that bulky hydrophobic residues are tolerated by the GLP-1R at position 10 and 11 (structure of **1**, ‘Introduction’), (2′-Et, 4′-OMe)BIP at position 10 was substituted by a SiFA moiety with concomitant variation of the α-quaternary amino acid at position 6 (Table 1). Both variants were synthesized to evaluate the effect of SiFA at position 10 and hence, their suitability as small peptide agents directly usable for ^18^F-labeling.

**Fig. 3:**
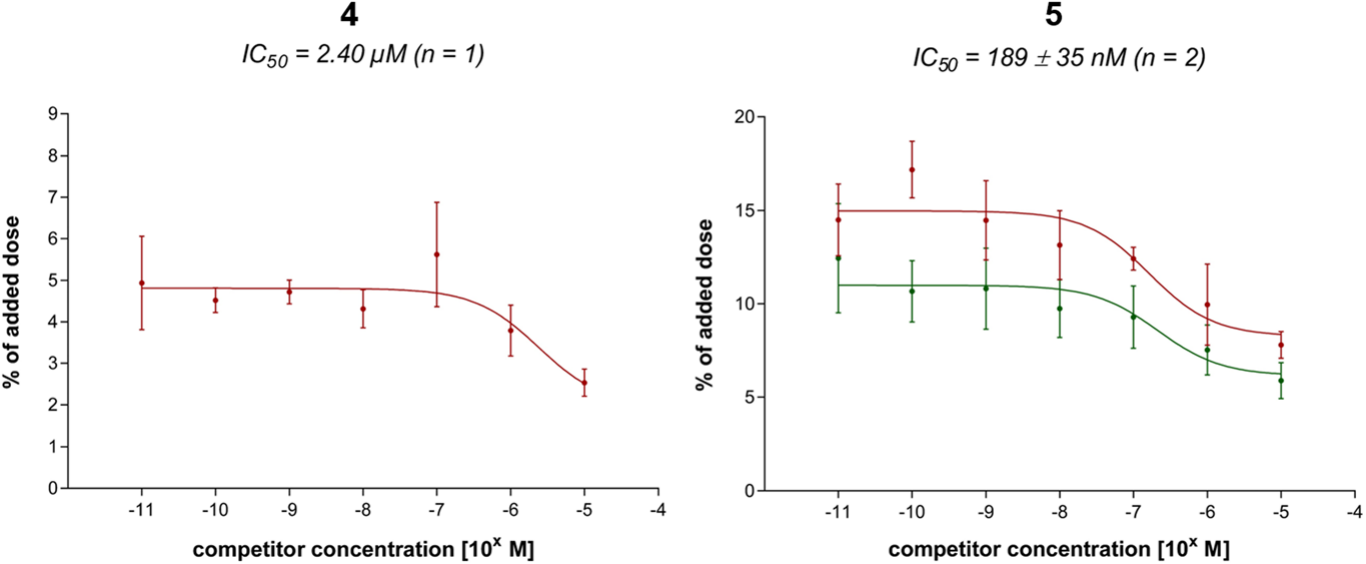
Sigmoidal dose-response curves for compounds **4** and **5** in 250 μL HBSS using HEK293-hGLP-1R cells (150,000 cells/well, 4 °C, 2 h) and [Nle^14^, [^125^I]Tyr(3-I)^40^]exendin-4 (0.41 nM) as radioligand

However, initial competitive binding experiments with bovine serum albumin (BSA) in the incubation buffer gave no detectable affinity neither for **4** nor for **5**. Due to the presence of the lipophilic SiFA moiety (Bernard-Gauthier et al., 2014), experiments were repeated in assay buffer (HBSS) without bovine serum albumin (BSA) as additive. Thereby, lipophilicity-induced nonspecific adhesion and retention by BSA should be avoided (Lexa et al., 2014). The curves of displacement indicated IC_50_ values of 2.40 μM (*n* = 1) for **4** and 189 ± 35 nM (*n* = 2) for **5**, though still high unspecific binding could be observed (maximal radioligand displacement 61–69% of maximal radioligand binding, Fig. 3).

In general, a positive trend towards **5** could be determined indicating more favorable properties for undecapeptides with (α-Me)Phe(2-F) instead of (α-Me)Phe as α-quaternary amino acid at position 6. A value of ~ 13 was obtained for the IC_50_ ratio **4**/**5** and thus confirmed the results obtained by Mapelli et al. (factor ~ 3.2) (Mapelli et al., 2009) to some extent, despite the higher factor determined within this study.

However, both peptides revealed only low ability to displace radioligand [^125^I]Tyr(3-I)^40^-**3** from HEK293-hGLP-1R cells and notably surpassed IC_50_ values of GLP-1 (23.2 ± 12.2 nM, *n* = 11) and [Nle^14^, Tyr(3-I)^40^]exendin-4 (7.63 ± 2.78 nM, *n* = 6). Hence, further undecapeptides containing a sterically demanding *C*-terminal dipeptide (L-Dap(SiFA)^10^-L-homoPhe^11^-NH_2_) were not synthesized. From these initial results it could not be deduced that undecapeptides with bulky substituents at the *C*-terminus would generally provide positive results, i.e. high affinity ligands. Therefore, the focus was shifted to examine if bulky substituents are mandatory or even harmful for receptor binding.

### Peptides without C-terminal bulky substituents

The *N*-terminal fragments of **4** and **5,** represented by **6** and **7** respectively, were investigated but exhibited no detectable affinity (Table 1). Even introduction of an amidated *C-*terminus in **7** installed for mimicking resemblance to a true peptide bond, did not result in any benefit. Likewise, substitution of the L-Dap(SiFA) moiety of **4** by a simple L-Phe(4-I) residue (**8**) eliminated any detectable affinity. Obviously, this hydrophobic residue cannot compensate for the sterically demanding SiFA-moiety as in **4** (IC_50_ = 2.40 μM). Consequently, the *C*-terminal dipeptide of lead peptide **1** or at least a hydrophobic bulky substituent at position 10 seemed to be indispensable for radioligand displacement within the 10^− 11^ to 10^− 5^ M range.

Since a certain tolerance, and especially for undecapeptides a mandatory need for bulky substituents emerged, SiFA-bearing peptides similar to **1** were further investigated, however, with structures closer related to the endogenous ligand GLP-1. Thereby, an increased affinity should be reached since the peptides investigated so far revealed IC_50_ values not below 189 ± 35 nM (*n* = 2) and therefore were not able to compete with high affinity GLP-1R-targeting peptides like GLP-1 (**2**) and [Nle^14^, Tyr(3-I)^40^]exendin-4 (Tyr(3-I)^40^–**3**).

### SiFA-tagged chimeras of 1 and GLP-1

Expanding the structural range, peptides with different chain lengths (11–15 amino acids) were synthesized and GLP-1-inherent amino acids were placed at the appropriate positions.

In order to avoid detrimental effects of the SiFA unit on ligand receptor interactions of the crucial *N*-terminal nonapeptide, repositioning towards the *C*-terminus was pursued. However, introduction of a SiFA moiety at position 11 was not tolerated, as no affinity was detected for peptide **9**.

By contrast, compounds with SiFA at position 13 like in **10**, **11** and **12** displayed affinities in the micromolar range (2.95 ± 0.31 μM to 348 ± 117 μM, Table 1). Indeed, an aromatic tyrosine residue can be found at this position in endogenous GLP-1, indicating a certain tolerance for this structural modification. It is important to note that further variations from original GLP-1 residues between Asp^9^ and Dap(SiFA)^13^ seem to be not well tolerated in those peptides, although no crucial role for receptor binding and activation could be ascribed by previous SAR studies (Gallwitz et al., 1994; Adelhorst et al., 1994). Substitution of L-Ser^11^ by a L-homoPhe residue as realized in peptide **1**, resulted in a ~ 2.4-fold decline in affinity (2.95 ± 0.31 μM for **10** vs. 7.12 ± 2.47 μM for **11**). Consequently, L-homoPhe at position 11 might be advantageous for receptor-ligand-interactions in undecapeptides like **1**, but obstructive if incorporated at the same position in extended peptides like **11**.

Furthermore, affinity also remarkably decreased by introduction of a short hydrophilic poly (ethylene oxide)-similar linker (O2Oc), which was installed in peptides **12** and **13** (Table 1). First, this linker fragment was supposed to compensate to a certain extent for the overall lipophilic character of the SiFA unit and was therefore introduced adjacent to the SiFA moiety in **12** and **13** (Israelachvili, 1997). Whereas peptide **12** showed low but still detectable affinity (348 ± 117 μM), no radioligand displacement was observed for its D-stereoisomer **13**. These results indicate that the natural L-configuration at position 13 might be preferred. Moreover, a certain rigidity introduced by the original GLP-1 residues Val^10^, Ser^11^ and Ser^12^ seems to be indispensable, since pentadecapeptides **10** and **11** still showed higher affinities (2.95 ± 0.31 μM and 7.12 ± 2.47 μM) compared to the more flexible O2Oc-modified analog **12** (348 ± 117 μM). Pentadecapeptides **10**, **11**, **12** and **13** were synthesized until Glu^15^ (Glu^21^ in GLP-1 counting method) for positioning a hydrophilic, anionic residue in close spatial proximity to the SiFA unit for partial compensation of the rather hydrophobic character.

### C-terminal pentadecapeptide of GLP-1

The importance of the C-terminal fragment of GLP-1 for receptor binding and selectivity was already proven by several studies (Hjorth et al., 1994; Underwood et al., 2010; Wu et al., 2020; Hoare, 2005). Based on these findings and the rather poor results obtained so far with *N*-terminal fragments of GLP-1 and derivatives thereof, a potential usability of the 15-residue *C*-terminal fragment of GLP-1 was investigated. Interestingly the isolated, acetylated *C*-terminal half of GLP-1 (**14**) exhibited a low but noticeable affinity (2.54 ± 0.70 μM, Table 1). However, expedient optimizations of this pentadecapeptide would be necessary to obtain a basic structure that tolerates modifications like chelator or SiFA attachment whilst keeping high affinity towards GLP-1R. For now, the IC_50_ of **14** exceeds the IC_50_ of GLP-1 by a factor of 109.

### Critical data analysis

It has to be noted that during the synthesis of compound **12**, racemization was detected after the coupling of Fmoc-L-Dap(Dde)-OH and resulted in an enantiomeric ratio (*er*) of ~ 3/7 for **12**/**13**. Peptides **12** and **13** were isolated by collecting the respective fractions separately (t_*R*_ = 6.81 min for **12** and t_*R*_ = 9.39 min for **13**, 80–100% Method B*, 1 mL/min. *Method B: solvent A = water + 0.1% TFA, solvent B = acetonitrile + 5% water + 0.1% TFA) via semi-preparative HPLC. Based on the following affinity determinations, peptide **12** was assumed to represent the stereoisomer comprising L-Dap(SiFA)^13^, since the natural ligand GLP-1 is assembled exclusively of L-amino acids. Further endeavors to determine the exact enantiomeric identity of compounds **12** and **13** were not undertaken, since even the favored enantiomer had a very low affinity (348 ± 117 μM). For all other compounds, no racemization was observed.

### Lead peptide 1

In previous studies, GLP-1R binding affinities of **1** and related derivatives either remained undefined, or were not compared to established ligands, such as GLP-1 or exendin-4 (Mapelli et al., 2009; Haque et al., 2010a; Haque et al., 2010b; Hoang et al., 2015; Swedberg et al., 2015). Therefore, the affinity of this GLP-1 mimetic should be determined in cell-based assays and compared to the values of known high affinity ligands GLP-1 and exendin-4. In vitro evaluation of **1** revealed an unfavorable IC_50_ value of 669 ± 242 nM (*n* = 5), which was not expected due to the very close EC_50_ of **1** to endogenous GLP-1 (EC_50_(**1**) = 31 pM, EC_50_(GLP-1) = 34 pM), as determined by Mapelli et al. and Haque et al. (Mapelli et al., 2009; Haque et al., 2010a)

Among all GLP-1R-targeting ligands, only **5** provided distinctly lower IC_50_ values (189 ± 35 nM, *n* = 2) than lead peptide **1** (669 ± 242 nM, n = 5). Although this suggests a higher GLP-1R affinity for **5**, nonspecific binding of this SiFA-bearing compound was notably high as displayed by the respective dose-response curves (Fig. 3). Introduction of charged amino acid residues (positive and/or negative) or other lipophilicity-reducing auxiliaries in close spatial proximity to the SiFA moiety might compensate for lipophilicity-induced unspecific binding of **5** (Bernard-Gauthier et al., 2014; Lexa et al., 2014). Accordingly, competitive binding studies with these ligands may provide more reliable IC_50_ values.

All in all, the rather poor results obtained for short-chained GLP-1 receptor ligands unveiled a huge discrepancy between the high potency of these ligands known from the literature and their actual low affinities, determined in HEK293-hGLP-1R cell-based assays. Especially **1** was supposed to possess a high affinity towards GLP-1R positive cells due to its inherent low EC_50_ of 31 pM determined by Haque et al. (Haque et al., 2010a). However, **1** lost its potential as a basic structure for novel promising small peptide GLP-1R agonists, since affinity studies revealed an unfavorable IC_50_ of 669 ± 242 nM (*n* = 5). It also remains questionable if small peptide approaches would generally provide high-affinity ligands, since previous SAR studies suggested that non-contiguous residues within the *N*- and *C*-terminus of GLP-1 and also in between were essential for high affinity and potency (Parker et al., 1998; Hjorth et al., 1994). As a consequence, yet synthesized SiFA-bearing ligands were not subjected to radiofluorination and no internalization or other in vitro and in vivo experiments were conducted due to the poor IC_50_ data generated by these compounds.

## Conclusions

In sum, the investigated structures provide no advantage over GLP-1- and exendin-4-based ligands. Only SiFA-tagged peptide **5** showed a higher affinity compared to **1**, but still not in a range which allows for in vivo evaluation. In consequence, radioligands targeting GLP-1R^+^ tumor lesions with reduced kidney uptake and/or enhanced renal excretion could not be generated. In order to reach an improved tumor localization and enable targeted radiotherapy of malignant insulinomas as well as of other GLP-1R-overexpressing malignancies, further investigations on pharmacokinetically optimized peptides should be envisaged.

## Supplementary Information

**Additional file 1.** Supporting Information is provided in addition to data presented in the main manuscript, including detailed information on all methods for synthesis and analysis as well as on the used instruments. Furthermore, procedures for ligand synthesis, analytical data and methods for in vitro characterization are given in more detail.

## Data Availability

The datasets supporting the conclusions of this article are included within this article and its additional file.

## Abbreviations

2-CT: 2-chlorotrityl
cAMP: 3′, 5′-cyclic adenosine monophosphate
A_m_: Molar activity
BIP: 4, 4′-L-biphenylalanine
BSA: Bovine serum albumin
CT: Computed tomography
Dap: 2,3-diaminopropionic acid
DCM: Dichloromethane
Dde: *N*-1-(4,4-dimethyl-2,6-dioxocyclohex-1-ylidene)ethylamine
DIC: N,N′-diisopropylcarbodiimide
DIPEA: *N*,*N*-diisopropylethylamine
DMA: *N,N*-dimethylacetamide
DMEM: Dulbecco’s Modified Eagle’s Medium
DMF: Dimethylformamide
DMSO: Dimethyl sulfoxide
EC_50_: Half maximal effective concentration
*er*: enantiomeric ratio
Fmoc: Fluorenylmethyloxycarbonyl
GLP-1: Glucagon-like peptide 1
GLP-1R: Glucagon-like peptide 1 receptor
GPCR: G protein-coupled receptor
HBSS: Hank’s buffered salt solution
HEK293: Human embryonic kidney 293 cells
HFIP: 1,1,1,3,3,3-hexafluoro-2-propanol
hGLP-1R: Human glucagon-like peptide 1 receptor
HOAt: 1-hydroxy-7-azabenzotriazole
IC_50_: Half maximal inhibitory concentration
MEN I: Multiple endocrine neoplasia type I
O2Oc: 8-amino-3,6-dioxaoctanoic acid
PBS: Phosphate-buffered saline
PET: Positron emission tomography
PPh_3_: Triphenylphosphane
PRRT: Peptide receptor radionuclide therapy
PyBOP: Benzotriazol-1-yl-oxytripyrrolidinophosphonium hexafluorophosphate
RACM: Rink amide ChemMatrix®
RCP: Radiochemical purity
RCY: Radiochemical yield
RP-HPLC: Reversed-phase high-performance liquid chromatography
r.t.: room temperature
SI: Supporting information
SiFA: Silicon-based fluoride acceptor
SPECT: Single-photon emission computed tomography
SPPS: Solid-phase peptide synthesis
TFA: Trifluoroacetic acid
TIPS: Triisopropylsilane
t_*R*_: retention time
