# Supplementary material for: Small peptide-based GLP-1R ligands: an approach to reduce the kidney uptake of radiolabeled GLP-1R-targeting agents?
Source: EJNMMI Radiopharm Chem. 2021 Aug 25;6:29. doi: 10.1186/s41181-021-00136-x (PMC8387526; doi:10.1186/s41181-021-00136-x)
Supplement: Supplementary file 1 — Additional file 1. Supporting Information is provided in addition to data presented in the main manuscript, including detailed information on all methods for synthesis and analysis as well as on the used instruments. Furthermore, procedures for ligand synthesis, analytical data and methods for in vitro characterization are given in more detail. [file 41181_2021_136_MOESM1_ESM.docx]

**Small peptide-based GLP-1R ligands: An approach to reduce the kidney uptake of radiolabeled GLP-1R-targeting agents?**

**- Supporting Information -**

Veronika Barbara Felber *, Hans-Jürgen Wester

*Chair of Pharmaceutical Radiochemistry, Technical University of Munich, Garching, Germany;*

**Institutional address of all authors:**

Technical University of Munich,

Chair of Pharmaceutical Radiochemistry,

Walther-Meißner-Str. 3

85748 Garching

GERMANY

***Corresponding author:**

Veronika Barbara Felber

Phone: +49.89.289.12237

Fax: +49.89.289.12204

Email: vroni.felber@tum.de

Keywords: GLP-1R, small peptides, insulinoma, diagnosis, peptide receptor radionuclide therapy, kidneys

1. GENERAL INFORMATION

All reagents were purchased from Merck KGaA (Darmstadt, Germany), Sigma-Aldrich Chemie GmbH (Steinheim, Germany), VWR International GmbH (Darmstadt, Germany), TCI (Eschborn, Germany), Iris Biotech (Marktredwitz, Germany) and Carbolution (St. Ingbert, Germany) in the quality grade “for synthesis”. [Nle^14^, Tyr^40^]Exendin-4 (**3**) and [Nle^14^, Tyr(3-I)^40^]exendin-4 (Tyr(3-I)^40^-**3**) were purchased from Biotrend Chemikalien GmbH (Cologne, Germany). Cell culture media and buffer solutions were purchased from Merck KGaA (Darmstadt, Germany) and Sigma Aldrich Chemie GmbH (Steinheim, Germany). ([^125^I]NaI was purchased from Hartmann Analytic (Braunschweig, Germany). Solvents were purchased from VWR International GmbH (Darmstadt, Germany) in the quality grade “HPLC grade”. Dry solvents were purchased from Sigma-Aldrich Chemie GmbH (Steinheim, Germany), Alfa Aesar (Karlsruhe, Germany) and VWR International GmbH (Darmstadt, Germany). Solid phase synthesis of the peptides was carried out by manual operation using an Intelli-Mixer syringe shaker (Neolab, Heidelberg, Germany).

1. MATERIALS

*Analytical* and *preparative* RP-HPLC were performed using Shimadzu gradient systems (Shimadzu Deutschland GmbH, Neufahrn, Germany), each equipped with a SPD-20A UV/Vis detector (220 nm, 254 nm). All systems were operated by the LabSolutions software. Prior to quality control acquisitions, a control run was performed, in which only water/acetonitrile (1/1) was injected. Thereby, the system was checked for impurities in the injection port or on the column.

As different eluents and flow rates have been used for several compounds, the used methods are cited in the text and described as follows:

Method A: solvent A = water + 0.1% TFA, solvent B = acetonitrile + 2% water + 0.1% TFA

Method B: solvent A = water + 0.1% TFA, solvent B = acetonitrile + 5% water + 0.1% TFA

Method C: solvent A = water, solvent B: acetonitrile

*Analytical RP-HPLC* was performed on a Nucleosil 100-C18 (5 μm, 125 mm x 4.6 mm) column (CS GmbH, Langerwehe, Germany) applying different linear solvent gradients (Method A) and a constant flow rate of 1 mL/min. Both, specific gradients and the corresponding retention times t*_R_* as well as capacity factor *k* are cited in the text. The capacity factor was calculated from the experimentally determined dead time (t_0_ = 1.6 min) of the HPLC system and the respective retention time t*_R_*:

$$k=\frac{t_{R}- t_{0}}{t_{0}}$$

*Preparative RP-HPLC* was performed on a Multospher 100 RP C18 (5 μm, 250 x 20 mm) column (CS GmbH, Langerwehe, Germany) applying different linear solvent gradients (Method B or C) and different constant flow rates of 5, 8, 9 or 10 mL/min.

*Flash chromatography* was performed with a Biotage gradient HPLC system (Biotage Europe, Uppsala, Sweden), using HP-Sphere C18-25 catridges (micron spherical silica, Biotage SNAP Ultra C18, 12 g). The compounds were eluted applying different solvent gradients (Method C).

*Radio-RP-HPLC* was performed on a Nucleosil 100-C18 (5 μm, 125 mm x 4.6 mm) column (CS GmbH, Langerwehe, Germany) using a Shimadzu gradient system (Shimadzu Deutschland GmbH, Neufahrn, Germany) with a linear solvent gradient (Method A) and a constant flow rate of 1 mL/min. For radioactivity detection, the outlet of the UV detector was connected to a HERM LB 500 NaI detector (Berthold Technologies, Bad Wildbad, Germany).

*Mass spectra* were acquired with an Advion expression L compact mass spectrometer (Advion Ltd., Harlow, UK) with electrospray ionization (positive ion mode) and an orthogonal ion sampling from the heated capillary. The system was operated by the Mass Express software and spectra were processed using the Data Express software.

*Activity measurements* of the respective probes obtained from competitive binding assays were measured by a 2480 Wizard^2^ automatic ɣ-counter (PerkinElmer, Waltham, USA) and evaluated with GraphPad PRISM7.

1. METHODS

General remarks on peptide synthesis

The used equivalents of the reactants for the solid phase synthesis refer to the calculated load after attaching the first amino acid onto the resin. The specific loads are cited in the text. Prior to any reaction, dry resin was swelled in NMP for at least 30 min and then filtered. Unless otherwise indicated, the resin was washed with DMF (6x) after each reaction step. For storage, the resin was washed with DMF (3x) and DCM (3x) and dried in a desiccator.

General procedure for loading the first amino acid onto Rink amide ChemMatrix^®^ resin and on-resin peptide bond formation (GP1)

Rink amide ChemMatrix^®^ resin (average load: 0.50 mmol/g) was obtained as the free *N*-terminal form and hence, loading of the first amino acid and further elongation steps were performed by the same standard procedure. The (first) amino acid/SiFA-BA (1.50 eq.), TBTU (1.50 eq.) and HOBt or HOAt (1.50 eq.) were dissolved in DMF (~ 10 mL/g resin) and preactivated by addition of DIPEA (4.50 eq.) for five minutes, prior to incubation with the resin. Unless otherwise noted, the solution was added to the resin and shaken for 2 h at room temperature. Occasionally, the pH value had to be adjusted to 9 ‑ 10 by addition of further DIPEA. In case of **11** and **12**, *sym*‑collidine (9.00 eq.) was used as base for coupling of Fmoc‑L‑Dap(Dde)-OH.

Independent of the resin (2-CT or RACM), coupling to resin-bound bulky α‑quaternary amino acids like H‑L‑(α‑Me)Phe and H‑L‑(α-Me)Phe(2-F) was performed with increased incubation periods (at least 24 h if bound to RACM or 2 × 17 ‑ 26 h in total if bound to 2-CT resin). Moreover, HATU instead of TBTU was used as coupling reagent and equivalents were scaled up to 3.00 eq. for the coupling amino acid (i.e. Fmoc‑L‑Thr(*t*Bu)-OH), HATU, HOBt or HOAt as well as to 5.00 ‑ 9.00 eq. for DIPEA (for a pH value of 9 ‑ 10). Coupling to resin-bound H-Aib was performed also with increased incubation periods (at least 5 h if bound to RACM or 24 h if bound to 2-CT resin). Additionally, equivalents of the coupling amino acid (i.e. Fmoc/Boc‑L‑His(Trt/Boc)-OH), TBTU, HOBt or HOAt (3.00 eq.) as well as for DIPEA (5.00 ‑ 9.00 eq.) were scaled up.

General procedure for loading the first amino acid onto 2-CTC resin (GP2)

The first amino acid (1.50 eq.) and DIPEA (1.33 eq.) were dissolved in DCM (5.00 mL) and stirred for 5 min at r.t. prior to addition of the resin (1.00 eq.). After 15 min further DIPEA (2.67 eq.) was added and the reaction mixture was stirred for 75 min. Afterwards, MeOH (2 mL) was added and stirred for 15 min. The resin was washed successively with MeOH (4x), DMF (4x) and DCM (4x) and dried at least two hours or overnight in a desiccator. The load was calculated using the following formula:

$$load=\frac{\left( m_{2}-m_{1} \right)\cdot1000}{{(M}_{W}-M_{\mathrm{HCl}}) \cdot m_{2}} \left[ \frac{\mathrm{mmol}}{g} \right]$$

M_W_ = molecular weight of the amino acid [g/mol]

M_HCl_ = molecular weight of HCl [g/mol]

*m_1_ =* mass of dry 2‑CTC resin before coupling [g]

*m_2_ =* mass of dried resin after coupling [g]

General procedure for on-resin peptide bond formation (GP3)

To a solution of TBTU (2.00 eq.), HOAt (2.00 eq.) and the amino acid (2.00 eq.) in DMF (~ 10 mL/g resin), DIPEA (6.00 - 9.00 eq.) was added to adjust the pH value to 9 ‑ 10 and the mixture was allowed to preactivate for five minutes. In the case of Fmoc‑D-Dap(Dde)-OH *sym*‑collidine (6.00 ‑ 8.00 eq.) was added instead of DIPEA. Unless otherwise noted, the solution was added to the resin-coupled peptide and shaken for 2 h at room temperature. Afterwards the resin was washed with DMF (6x).

General procedure for the on-resin Fmoc-removal (GP4)

The resin was shaken 5 × 5 min in 20% piperidine in DMF (v/v) to remove the Fmoc-protective group and afterwards washed with DMF (7x). If Ornithin was the first amino acid bound to the resin, Fmoc-removal was performed 12 x 5min in 20% piperidine.

General procedures for the on-resin Dde-removal (GP5 & GP6)

GP5: If no Fmoc-group was present in the resin bound peptide, the resin was treated with 2% hydrazine in

DMF (10 mL) for 20 min and afterwards washed with DMF (7x).

GP6: If an Fmoc-group was present in the resin bound peptide, the resin was treated with a solution of

imidazole (0.46 g/g resin) and hydroxylamine hydrochloride (0.63 g/g resin) in NMP (5.0 mL/g resin)

and DMF (1.0 mL/g resin) for 2 x 3 h. Afterwards, the resin was washed with DMF (7x).

General procedures for monitoring the reaction progress (GP7 & GP8)

For a test cleavage with TFA (GP7) a small aliquot of the resin was taken and treated with 100 μL of TFA for 15 min at r.t. in an Eppendorf tube.

To avoid cleavage of *tert*-butyl groups or formation of other unidentifiable by-products, test cleavage with HFIP/DCM (GP8) was used. The resin aliquot was treated with 100 μL of HFIP/DCM (1/4, v/v) for 30 min at r.t.

For both procedures the respective solution (without beads!) was transferred into another Eppendorf tube and the solvent was evaporated under a stream of nitrogen. The residue was dissolved in a mixture of H_2_O and MeCN (1/1, v/v), now ready for RP‑HPLC analysis.

Cleaving the peptide off the resin with simultaneous removal of all acid-labile protective groups (GP9)

The resin was treated with TFA/TIPS/DCM (95/2.5/2.5, 10.0 mL) once or twice for 30 min at r.t. and washed with DCM afterwards (3x). The solvent was evaporated under N_2_ flow and after lyophilisation the crude product was obtained.

Peptide cleavage from the resin with preservation of all acid-labile protective groups (GP10 & GP 11)

GP10: The resin was treated with DCM/TFE/AcOH (6/3/1, 10 - 12 mL) for 30 min at r.t and washed with DCM afterwards (6×). Solvents were removed under reduced pressure and AcOH was removed by azeotropic distillation (addition of small portions of toluene, 4×). After removal of residual toluene and lyophilization the crude product was obtained.

GP11**:** The resin was treated with HFIP/DCM (1/4, 10 mL) for 4 h (4 × 30 min, 2 × 1 h) at r.t. and washed with DCM afterwards (6×). Solvents were evaporated under N_2_ flow and after lyophilization the crude product was obtained.

- 1. Synthesis

*Glucagon-like peptide 1 (****2****)*

GLP-1 (**2**) was synthesized according to standard Fmoc‑SPPS on Rink amide ChemMatrix^®^ resin (0.20 mmol, 1.00 eq.), applying the above-mentioned methods (GP1 and GP4). The reaction progress was monitored (GP7) after coupling of Fmoc‑L‑Leu^32^-OH, Fmoc‑L‑Glu^27^(O*t*Bu)‑OH, Fmoc‑L‑Gln^23^(Trt)‑OH, Fmoc‑L‑Leu^20^-OH, Fmoc‑L‑Tyr^19^(O*t*Bu)-OH, Fmoc‑L‑Ser^14^(*t*Bu)-OH, Fmoc‑Gly^10^‑OH and Fmoc‑L‑His^7^(Trt)-OH. Formation of the expected products was confirmed by RP‑HPLC/MS and after removal of the Fmoc protective group from *N*‑terminal histidine, the peptide was cleaved off the resin with TFA/TIPS/H_2_O (95/2.5/2.5, 1 × 30 min, GP9). The solvent was reduced in a stream of nitrogen, the peptide was precipitated in Et_2_O and centrifuged (3000 rpm, ca. 1000 g, 3 min). Purification by preparative RP‑HPLC (35 ‑ 50% B in 20 min, Method B, 5 mL/min) was performed with a small portion of the precipitate and afforded 11.7 mg of pure product **2***5TFA as a colorless powder after lyophilization.

RP-HPLC (10 ‑ 90% B in 15 min, Method A, 1 mL/min): t*_R_* = 9.21 min; *k*= 5.1. Calculated monoisotopic mass (C_149_H_226_N_40_O_45_): 3295.66, found: *m/z* = 1649.0 [M(**2**)+2H]^2+^, 1099.7 [M(**2**)+3H]^3+^, 825.1 [M(**2**)+4H]^4+^.

*Fmoc‑L‑His(Trt)‑Aib‑L‑Glu(OtBu)-Gly‑L‑Thr(tBu)‑L‑(α-Me)Phe‑L‑Thr(tBu)‑L‑Ser(tBu)‑L‑Asp(OtBu)‑OH (****S‑1****)*

Nonapeptide **S‑1** was synthesized on 2‑CT resin according to GP2, GP3 and GP4 (load: 1.02 mmol/g, 0.31 mmol, 1.00 eq.). The peptide was cleaved off the resin according to GP10. Thereby, 545 mg of crude product **S‑1** were obtained and used in subsequent steps without further purification.

RP-HPLC (40 ‑ 100% B in 15 min, Method A, 1 mL/min): t*_R_*= 17.6 min; *k*= 11. Calculated monoisotopic mass (C_96_H_125_N_11_O_19_): 1735.92, found: *m/z* = 868.9 [M(**S‑1**)+2H]^2+^, 1495.4 [M(**S‑1**)‑Trt+H]^+^, 1737.3 [M(**S‑1**)+H]^+^.

*Fmoc‑L‑Dap(SiFA)‑L‑homoPhe-RACM (****S‑2****)*

Dipeptide **S‑2** was synthesized on Rink amide ChemMatrix^®^ resin according to GP1, GP4 and GP6 (load: 0.50 mmol/g, 0.15 mmol, 1.00 eq.). A test cleavage (GP7) was performed after coupling of SiFA‑BA, which revealed nearly complete conversion to product **S‑2**.

RP-HPLC (10 ‑ 90% B in 15 min, Method A, 1 mL/min): t*_R_*= 19.5 min; *k*= 12. Calculated monoisotopic mass (C_43_H_51_FN_4_O_5_Si): 750.36, found: *m/z* = 751.4 [M(**S‑2**)+H]^+^, 1501.9 [M_2_(**S‑2**)+H]^+^.

*H‑L‑His‑Aib‑L‑Glu-Gly‑L‑Thr‑L‑(α-Me)Phe‑L‑Thr‑L‑Ser‑L‑Asp‑L‑Dap(SiFA)‑L‑homoPhe-NH_2_ (****4****)*

Synthesis of peptide **4** was conducted via fragment coupling according to GP1 and GP4. First, the Fmoc protective group was removed from **S‑2** (37.5 µmol, 1.00 eq.) (GP4). Afterwards, a solution containing **S‑1** (97.2 mg, 56.3 µmol, 1.50 eq.), TBTU (24.1 mg, 75.0 μmol, 2.00 eq.), HOBt (10.1 mg, 75.0 μmol, 2.00 eq.) and DIPEA (28.7 μL, 169 μmol, 4.50 eq.) in DMF was added and incubated for 15 h at room temperature. Examination of the reaction progress (GP7) revealed successful coupling of **S‑1**. Removal of the *N*-terminal Fmoc protective group (GP4), followed by cleavage of the peptide from the resin with TFA/TIPS/H_2_O (95/2.5/2.5, 1 × 30 min, GP9) resulted in 2.70 mg (4.16%) of product **4***2 TFA as a colorless powder after RP-HPLC purification (35 ‑ 50% B in 20 min, Method B, 5 mL/min) and lyophilization.

RP-HPLC (10 ‑ 90% B in 15 min, Method A, 1 mL/min): t*_R_*= 10.6 min; *k*= 6.1. Calculated monoisotopic mass (C_70_H_100_FN_15_O_19_Si): 1501.71, found: *m/z* = 751.9 [M(**4**)+2H]^2+^, 1502.6 [M(**4**)+H]^+^.

*Fmoc‑L‑His(Trt)-Aib‑L‑Glu(OtBu)-Gly‑L‑Thr(tBu)‑L‑(α-Me)Phe(2‑F)‑L‑Thr(tBu)‑L‑Ser(tBu)-L‑Asp(OtBu)‑OH (****S-3****)*

Nonapeptide **S‑3** was synthesized on 2‑CT resin according to GP2, GP3 and GP4 (load: 1.38 mmol/g, 0.21 mmol, 1.00 eq.). The peptide was cleaved off the resin according to GP10. Thereby, 162 mg of crude product **S‑3** were obtained and used in subsequent steps without further purification.

RP-HPLC (40 ‑ 100% B in 15 min, Method A, 1 mL/min): t*_R_*= 17.5 min; *k*= 11. Calculated monoisotopic mass (C_96_H_124_FN_11_O_19_): 1753.91, found: *m/z* = 878.0 [M(**S‑3**)+2H]^2+^, 1513.7 [M(**S‑3**)‑Trt+H]^+^, 1754.6 [M(**S‑3**)+H]^+^.

*H‑L‑His-Aib‑L‑Glu-Gly‑L‑Thr‑L‑(α-Me)Phe(2-F)‑L‑Thr‑L‑Ser‑L‑Asp‑L‑Dap(SiFA)‑L‑homoPhe-NH_2_ (****5****)*

Synthesis of peptide **5** was conducted via fragment coupling according to GP1 and GP4. First, the Fmoc protective group was removed from **S-2** (37.5 µmol, 1.00 eq.) (GP4). Afterwards, a solution containing **S-3** (98.2 mg, 64.6 µmol, 1.72 eq.), TBTU (24.1 mg, 75.0 μmol, 2.00 eq.), HOBt (10.1 mg, 75.0 μmol, 2.00 eq.) and DIPEA (28.7 μL, 169 μmol, 4.50 eq.) in DMF was added and incubated for 15 h at room temperature. Examination of the reaction progress (GP7) revealed successful coupling of **S-3**. Removal of the *N*-terminal Fmoc protective group (GP4), followed by cleavage of the peptide from the resin with TFA/TIPS/H_2_O (95/2.5/2.5, 1 × 30 min, GP9) resulted in 5.40 mg (8.24%) of product **5***2 TFA as a colorless powder after RP-HPLC purification (35 ‑ 50% B in 20 min, Method B, 5 mL/min) and lyophilization.

RP-HPLC (10 ‑ 90% B in 15 min, Method A, 1 mL/min): t*_R_*= 10.6 min; *k*= 6.1. Calculated monoisotopic mass (C_70_H_99_F_2_N_15_O_19_Si): 1519.70, found: *m/z* = 761.4 [M(**5**)+2H]^2+^, 1520.9 [M(**5**)+H]^+^.

*H‑L‑His-Aib‑L‑Glu-Gly‑L‑Thr‑L‑(α-Me)Phe‑L‑Thr‑L‑Ser‑L‑Asp-OH (****6****)*

A small portion of hydrophobic nonapeptide **S‑1** (not weighed) was partitioned by salt‑induced precipitation in H_2_O and the lyophilized crude product was incubated with 1.10 mL TFA/TIPS (91/9) for 30 min. TFA was removed under a stream of nitrogen prior to addition of 3.00 mL 20% piperidine in DMF (v/v). After an incubation period of 20 min, crude product **6** was precipitated in Et_2_O and centrifuged (5300 rpm, ca. 3100 × g, 4 min, 20 °C). Purification by RP‑HPLC (10 ‑ 20% B in 20 min, Method B, 5 mL/min) afforded 7.60 mg of product **6***2 TFA as a colorless powder.

RP-HPLC (10 ‑ 90% B in 15 min, Method A, 1 mL/min): t*_R_*= 4.25 min; *k*= 1.8. Calculated monoisotopic mass (C_42_H_61_N_11_O_17_): 991.42, found: *m/z* = 496.9 [M(**6**)+2H]^2+^, 992.4 [M(**6**)+H]^+^.

*Boc‑L‑His(Boc)-Aib‑L‑Glu(OtBu)-Gly‑L‑Thr(tBu)‑L‑(α-Me)Phe(2-F)‑L‑Thr(tBu)‑L‑Ser(tBu)-L‑Asp(OtBu)‑OH (****19****)*

Nonapeptide **19** was synthesized on 2‑CT resin according to GP2, GP3 and GP4 (load: 1.05 mmol/g, 1.73 mmol, 1.00 eq.). The peptide was cleaved off the resin according to GP10. This afforded 2.72 g of crude product **19** as a slightly yellow solid, which was used in subsequent steps without further purification.

RP-HPLC (10 ‑ 90% B in 15 min, Method A, 1 mL/min): t*_R_*= 19.7 min; *k*= 12. Calculated monoisotopic mass (C_72_H_116_FN_11_O_21_): 1489.83, found: *m/z* = 1391.0 [M(**19**)-Boc+H]^+^, 1491.1 [M(**19**)+H]^+^.

*H‑L‑His-Aib‑L‑Glu-Gly‑L‑Thr‑L‑(α-Me)Phe(2-F)‑L‑Thr‑L‑Ser‑L‑Asp-NH_2_ (****7****)*

Crude product **19** (10.0 mg, ~ 6.71 µmol, 1.00 eq.) was coupled to Rink amide ChemMatrix^®^ resin according to GP1. After incubation for 2 h at room temperature, formation of the expected product was confirmed by RP-HPLC/MS (GP7) and hence, the now amidated peptide was cleaved off by TFA/TIPS/H_2_O (2 × 30 min, GP9). TFA was removed under a stream of nitrogen, H_2_O was added to the crude product, frozen (-80 °C) and lyophilized. Purification by RP‑HPLC (10 ‑ 60% B in 15 min, Method B, 1 mL/min) afforded 0.40 mg (4.82%) of product **7***2 TFA as a colorless powder.

RP-HPLC (10 ‑ 60% B in 15 min, Method A, 1 mL/min): t*_R_*= 8.38 min; *k*= 4.6. Calculated monoisotopic mass (C_42_H_61_FN_12_O_16_): 1008.43, found: *m/z* = 505.5 [M(**7**)+2H]^2+^.

*H‑L‑Phe(4-I)‑L‑homoPhe-RACM (****S‑4****)*

Dipeptide **S‑4** was synthesized on Rink amide ChemMatrix^®^ resin according to GP1 and GP4 (load: 0.50 mmol/g, 0.30 mmol, 1.00 eq.). After coupling of Fmoc‑L‑Phe(4-I)-OH (GP1) and removal of the *N*‑terminal Fmoc protective group, formation of the expected product was confirmed by RP‑HPLC/MS analysis (GP7), which revealed nearly complete conversion to product **S‑4**.

RP-HPLC (40 ‑ 100% B in 15 min, Method A, 1 mL/min): t*_R_*= 4.76 min; *k*= 2.2. Calculated monoisotopic mass (C_19_H_22_IN_3_O_2_): 451.08, found: *m/z* = 452.4 [M(**S-4**)+H]^+^.

*H‑L‑His-Aib‑L‑Glu-Gly‑L‑Thr‑L‑(α-Me)Phe‑L‑Thr‑L‑Ser‑L‑Asp‑L‑Phe(4-I)‑L‑homoPhe-NH_2_ (****8****)*

Synthesis of peptide **8** was conducted via fragment coupling according to GP1. A solution containing **S‑1** (617 mg, 0.35 mmol, 1.18 eq.), TBTU (193 mg, 0.60 mmol, 2.00 eq.), HOBt (81.1 mg, 0.60 mmol, 2.00 eq.) and DIPEA (230 μL, 1.35 mmol, 4.50 eq.) in DMF was added to *C*-terminal dipeptide **S‑4** (0.30 mmol, 1.00 eq.) and incubated for 22 h at room temperature. Examination of the reaction progress (GP7) revealed successful coupling of **S‑1**. Removal of the *N*-terminal Fmoc protective group (GP4), followed by cleavage of the peptide from the resin with TFA/TIPS/H_2_O (95/2.5/2,5, 1 × 30 min, slightly modified to GP9) resulted in 4.47 mg (0.90%) of product **8***2 TFA as a colorless powder after RP‑HPLC purification (35 ‑ 38% B in 20 min, Method B, 5 mL/min) and lyophilization.

RP-HPLC (10 ‑ 90% B in 15 min, Method A, 1 mL/min): t*_R_*= 8.25 min; *k*= 4.5. Calculated monoisotopic mass (C_61_H_81_IN_14_O_18_): 1424.49, found: *m/z* = 713.5 [M(**8**)+2H]^2+^, 1425.8 [M(**8**)+H]^+^.

*Fmoc‑L‑Val‑L‑Dap(SiFA)-RACM (****S‑5****)*

Dipeptide **S‑5** was synthesized on Rink amide ChemMatrix^®^ resin according to GP1, GP4 and GP6 (load: 0.50 mmol/g, 51.0 µmol, 1.00 eq.). After coupling of Fmoc‑L‑Val-OH, formation of the expected product was confirmed by RP‑HPLC/MS analysis (GP7), which revealed nearly complete conversion to product **S‑5**.

RP-HPLC (40 ‑ 100% B in 15 min, Method A, 1 mL/min): t*_R_*= 16.7 min; *k*= 10. Calculated monoisotopic mass (C_38_H_49_FN_4_O_5_Si): 688.35, found: *m/z* = 689.3 [M(**S‑5**)+H]^+^.

*H‑L‑His-Aib‑L‑Glu-Gly‑L‑Thr‑L‑(α-Me)Phe(2-F)‑L‑Thr‑L‑Ser‑L‑Asp‑L‑Val‑L‑Dap(SiFA)-NH_2_ (****9****)*

Synthesis of peptide **9** was conducted via fragment coupling according to GP1 and GP4. First, the Fmoc protective group was removed from **S‑5** (25.5 µmol, 1.00 eq.) (GP4). Afterwards, a solution containing **S‑3** (40.3 mg, 23.0 µmol, 0.90 eq.), TBTU (16.4 mg, 51.0 μmol, 2.00 eq.), HOBt (6.89 mg, 51.0 μmol, 2.00 eq.) and DIPEA (19.6 μL, 115 μmol, 4.50 eq.) in DMF was added and incubated for 24 h at room temperature. Examination of the reaction progress (GP7) revealed successful coupling of **S‑3**. Removal of the *N*-terminal Fmoc protective group (GP4), followed by cleavage of the peptide from the resin with TFA/TIPS/H_2_O (1 × 30 min, GP9) resulted in 8.40 mg (21.7%) of product **9***2 TFA as a colorless powder after RP-HPLC purification (30 ‑ 80% B in 20 min, Method B, 5 mL/min) and lyophilization.

RP-HPLC (10 ‑ 90% B in 15 min, Method A, 1 mL/min): t*_R_*= 9.59 min; *k*= 5.4. Calculated monoisotopic mass (C_65_H_97_F_2_N_15_O_19_Si): 1457.68, found: *m/z* = 730.2 [M(**9**)+2H]^2+^, 1458.9 [M(**9**)+H]^+^.

*H‑L‑His-Aib‑L‑Glu-Gly‑L‑Thr‑L‑(α-Me)Phe(2-F)‑L‑Thr‑L‑Ser‑L‑Asp‑L‑Val‑L‑Ser‑L‑Ser‑L‑Dap(SiFA)- L‑Leu‑L‑Glu-NH_2_ (****10****)*

Preparation of pentadecapeptide **10** was conducted via linear synthesis on Rink amide ChemMatrix^®^ resin according to GP1, GP4 and GP6 (load: 0.50 mmol/g, 0.13 mmol, 1.00 eq.). After coupling of Boc‑L‑His(Boc)‑OH, formation of the expected product was confirmed by RP‑HPLC/MS analysis (GP7). Cleavage of the peptide from the resin with TFA/TIPS/DCM (2 × 30 min, GP9) resulted in 22.2 mg (8.12%) of pure product **10***2 TFA as a colorless powder after RP‑HPLC purification (40 ‑ 70% B in 20 min, Method B, 5 mL/min) and lyophilization.

RP-HPLC (10 ‑ 90% B in 15 min, Method A, 1 mL/min): t*_R_*= 10.2 min; *k*= 5.8. Calculated monoisotopic mass (C_82_H_125_F_2_N_19_O_27_Si): 1873.87, found: *m/z* = 937.8 [M(**10**)+2H]^2+^, 1250.4 [M_2_(**10**)+3H]^3+^, 1875.2 [M(**10**)+H]^+^.

*H‑L‑His-Aib‑L‑Glu-Gly‑L‑Thr‑L‑(α-Me)Phe(2-F)‑L‑Thr‑L‑Ser‑L‑Asp‑L‑Val‑L‑homoPhe‑L‑Ser-L‑Dap(SiFA)‑L‑Leu‑L‑Glu-NH_2_ (****11****)*

Preparation of pentadecapeptide **11** was conducted via linear synthesis on Rink amide ChemMatrix^®^ resin according to GP1, GP4 and GP6 (load: 0.50 mmol/g, 0.13 mmol, 1.00 eq.). After coupling of Boc‑L‑His(Boc)‑OH, formation of the expected product was confirmed by RP‑HPLC/MS analysis (GP7). Cleavage of the peptide from the resin with TFA/TIPS/DCM (2 × 30 min, GP9) resulted in 17.2 mg (6.08%) of pure product **11***2 TFA as a colorless powder after RP‑HPLC purification (40 ‑ 70% B in 20 min, Method B, 5 mL/min) and lyophilization.

RP-HPLC (10 ‑ 90% B in 15 min, Method A, 1 mL/min): t*_R_*= 11.9 min; *k*= 6.9. Calculated monoisotopic mass (C_89_H_131_F_2_N_19_O_26_Si): 1947.92, found: *m/z* = 974.7 [M(**11**)+2H]^2+^, 1299.6 [M_2_(**11**)+3H]^3+^, 1949.1 [M(**11**)+H]^+^.

*H-O2Oc-L/D-Dap(SiFA)‑L‑Leu‑L‑Glu(OtBu)-RACM (****S‑6****)*

Peptide **S‑6** was synthesized on Rink amide ChemMatrix^®^ resin according to GP1, GP4 and GP6 (load: 0.50 mmol/g, 0.13 mmol, 1.00 eq.). After coupling of 8‑(9-Fmoc)amino-3,6-dioxaoctanoic acid and removal of the *N*-terminal Fmoc protective group, the expected product was confirmed by RP‑HPLC/MS analysis (GP7), which revealed nearly complete conversion to product **S‑6**.

RP-HPLC (10 ‑ 90% B in 15 min, Method A, 1 mL/min) for **S‑6(-*t*Bu)**: t*_R_*= 15.5 min; *k*= 9.3. Calculated monoisotopic mass for **S‑6(-*t*Bu)** (C_35_H_59_FN_6_O_9_Si): 754.41, found: *m/z* = 797.6 [M(**S‑6**)‑*t*Bu+MeCN+H]^+^, 1595.4 [M_2_(**S‑6**)-*t*Bu+MeCN)+H]^+^.

*H‑L‑His-Aib‑L‑Glu-Gly‑L‑Thr‑L‑(α-Me)Phe(2-F)‑L‑Thr‑L‑Ser‑L‑Asp-O2Oc-L/D-Dap(SiFA)‑L‑Leu‑L‑Glu-NH_2_ (****12****/****13****)*

Synthesis of peptides **12** and **13** was conducted via fragment coupling of the *N*-terminal fragment **19** with the resin-bound *C*-terminal peptide **S‑6** according to GP1. A solution containing **19** (99.3 mg, 66.7 µmol, 1.00 eq.), TBTU (32.1 mg, 66.7 µmol, 1.00 eq.), HOAt (13.6 mg, 66.7 µmol, 1.00 eq.) and DIPEA (51.0 μL, 300 μmol, 4.50 eq.) in DMF was added and incubated for 3 h at room temperature. Examination of the reaction progress (GP7) revealed only very low conversion, hence **19** and all further reagents were again weighed, added and coupling was conducted for 29 h. After this period RP‑HPLC/MS analysis (GP7) revealed sufficient conversion. Cleavage of the peptide from one half of the resin was conducted with pure TFA (1 × 30 min, slightly modified to GP9). Purification by RP-HPLC (40 ‑ 90% B in 30 min, Method B, 5 mL/min and 80 ‑ 100% B in 15 min, Method B, 1 mL/min) and lyophilization resulted in 1.41 mg (2.14%) of pure product **12***2 TFA and 2.38 mg (3.62%) of pure product **13***2 TFA, both as colorless powder.

Racemization occurred during the coupling of Fmoc-L‑Dap(Dde)-OH for synthesis of **S‑6** and led to an enantiomeric ratio (*er*) of ~ 3/7 for **12**/**13**. The identity of the respective enantiomers was assigned retrospectively by IC_50_ studies of **12** and **13**.

**12**: RP-HPLC (80 ‑ 100% B in 15 min, Method B, 1 mL/min): t*_R_*= 6.81 min; *k*= 3.5. Calculated monoisotopic mass (C_77_H_117_F_2_N_17_O_25_Si): 1745.81, found: *m/z* = 875.1 [M(**12**)+2H]^2+^, 1747.8 [M(**12**)+H]^+^.

**13**: RP-HPLC (80 ‑ 100% B in 15 min, Method B, 1 mL/min): t*_R_*= 9.39 min; *k*= 5.3. Calculated monoisotopic mass (C_77_H_117_F_2_N_17_O_25_Si): 1745.81, found: *m/z* = 874.6 [M(**13**)+2H]^2+^, 1748.7 [M(**13**)+H]^+^.

*Acetyl-Gly‑L‑Gln‑L‑Ala‑L‑Ala‑L‑Lys‑L‑Glu‑L‑Phe‑L‑Ile‑L‑Ala‑L‑Trp‑L‑Leu‑L‑Val‑L‑Lys-Gly‑L‑Arg-NH_2_ (****14****)*

Preparation of pentadecapeptide **14** was conducted via linear synthesis on Rink amide ChemMatrix^®^ resin according to GP1 and GP4 (load: 0.50 mmol/g, 93.3 µmol, 1.00 eq.). After coupling of Ac-Gly-OH, formation of the expected product was confirmed by RP‑HPLC/MS analysis (GP7). Cleavage of the peptide from the resin with TFA/TIPS/H_2_O (1 × 30 min, GP9) resulted in 10.2 mg (5.32%) of pure product **14***3 TFA as a colorless powder after RP-HPLC purification (40 ‑ 55% B in 20 min, Method B, 10 mL/min) and lyophilization.

RP-HPLC (10 ‑ 90% B in 15 min, Method B, 1 mL/min): t*_R_*= 11.1 min; *k*= 6.4. Calculated monoisotopic mass (C_80_H_127_N_23_O_19_): 1713.97, found: *m/z* = 573.1 [M_3_(**14**)+9H]^9+^, 858.6 [M(**14**)+2H]^2+^, 1144.0 [M_2_(**14**)+3H]^3+^, 1716.0 [M(**14**)+H]^+^.

*Boc‑L‑(2’-Et, 4’-OMe)BIP‑L‑homoPhe-NH_2_ (****17****)*

Compound **17** was synthesized in analogy to a previously published procedure by Haque *et al.*^(1)^ with some minor modifications. Resin-bound Boc*‑*L*‑*Phe(4-I)-2-CT (**15**) (load: 0.92 mmol/g, 0.56 mmol, 1.00 eq.) was transferred to a round-bottom flask. Boronic acid **16** (400 mg, 2.22 mmol, 4.00 eq.) dissolved in 4.77 mL DMA, Pd(PPh_3_)_4_ (64.7 mg, 56.0 µmol, 0.10 eq.) dissolved in 1.60 mL DMA (6.37 mL DMA in total) and 2.24 mL of an aqueous 2 M K_3_PO_4_ solution (4.48 mmol, 8.00 eq.) were added to the resin. The yellow-orange solution was heated to 80 °C and stirred under argon for 22 h. Afterwards, the resin was transferred to a syringe equipped with a frit, pore size 25 µm) and washed once with DMF. This fraction was kept together with the reaction solution. The resin was washed alternating with 10 mL of 0.5% (w/v) DDTC in DMF (3×) and 10 mL of 0.5% (v/v) DIPEA in DMF (3×) to remove palladium catalyst and other cross-coupling reagents from the resin. After final washing steps with DMF (3×) and DCM (3×) formation of product **17** could be confirmed on the resin by RP‑HPLC/MS analysis (GP7). Besides, the major part of product **17** was found in the reaction solution. Therefore, it was either directly purified by flash chromatography (20 ‑ 80% B in 10 min, Method C, 12 mL/min) or by preparative RP‑HPLC (20 ‑ 80% B in 20 min, Method B, 5 mL/min) after incubation of the resin with HFIP/DCM (GP11). In this case, the latter yielded 1.36 mg (0.61%) of purified product **17** as a brown-orange viscous oil.

RP-HPLC (20 ‑ 80% B in 15 min, Method A, 1 mL/min): t*_R_*= 17.7 min; *k*= 11. Calculated monoisotopic mass (C_23_H_29_NO_5_): 399.20, found: *m/z* = 300.2 [M(**17**)-Boc+H]^+^, 341.2 [M(**17**)‑Boc+MeCN+H]^+^, 344.2 [M(**17**)-*t*Bu+H]^+^, 385.2 [M(**17**)-*t*Bu+MeCN]^+^.

*H‑L‑(2’-Et, 4’-OMe)BIP‑L‑homoPhe-NH_2_ (****18****)*

Compound **18** was synthesized in analogy to a previously published procedure by Haque *et al.*^(2)^ with some minor modifications. Resin‑bound H*‑*L‑homoPhe-RACM (load: 0.39 mmol/g, 56.4 µmol, 1.00 eq.) was swelled in NMP for 15 min. Meanwhile, **17** (28.9 mg, 73.3 µmol, 1.30 eq.) was dissolved in 3 mL DMF/DCM (2/1) and added to PyBOP (38.1 mg, 73.3 µmol, 1.30 eq.) and HOAt (10.0 mg, 73.3 µmol 1.30 eq.). DIPEA (72.3 µL, 0.43 mmol, 7.54 eq.) was added and the reaction mixture was incubated with H‑L‑homoPhe‑RACM for 24 h. Examination of the reaction progress (GP7) revealed successful conversion to product **18**, which led to cleavage of the dipeptide from the resin with TFA/TIPS/H_2_O (1 × 30 min, GP9). After removal of TFA under a stream of nitrogen, H_2_O was added to the crude product, frozen (-80 °C) and lyophilized. Purification by RP-HPLC (30 ‑ 90% B in 20 min, Method B, 5 mL/min) afforded 2.94 mg (9.09%) of product **18***TFA as a colorless powder.

RP-HPLC (40 ‑ 100% B in 15 min, Method A, 1 mL/min): t*_R_*= 8.27 min; *k*= 4.5. Calculated monoisotopic mass (C_28_H_33_N_3_O_3_): 459.25, found: *m/z* = 460.4 [M(**18**)+H]^+^, 482.4 [M(**18**)+Na]^+^, 498.4 [M(**18**)+K]^+^.

*Boc‑L‑His(Boc)-Aib‑L‑Glu(OtBu)-Gly‑L‑Thr(tBu)‑L‑(α-Me)Phe(2-F)‑L‑Thr(tBu)‑L‑Ser(tBu)‑L‑Asp(OtBu)-L‑(2’‑Et, 4’‑OMe)BIP‑L‑homoPhe-NH_2_ (****S‑7****)*

Compound **S‑7** was synthesized in analogy to a previously published procedure by Haque *et al.*^(2)^ with some minor modifications. First, the counterion TFA was removed from **18** (1.42 mg, 2.48 µmol, 1.00 eq.) by tetraalkylammonium carbonate (macroporous, polymer-bound, 18 ‑ 50 mesh, average load = 3.00 mmol/g). Therefore, **18***TFA was dissolved in 300 µL THF (supplemented with 0.5% DIPEA (v/v)), the anion exchange resin (2.48 mg, 7.43 µmol, 3.00 eq.) was added and the mixture was stirred in a sealed glass vial for 2 h at room temperature. The reaction solution was separated from the resin and the solvent was removed *in vacuo*. Nonapeptide **19** (3.69 mg, 2.48 µmol, 1.00 eq.), HOAt (0.67 mg, 4.95 µmol, 2.00 eq.) and DIC (3.07 µL, 19.7 µmol, 8.00 eq.) were dissolved in 510 µL DCM/DMF (9/1) and added to **18**. If necessary, DIPEA was added to adjust the pH to 9 ‑ 10 and the mixture was stirred for 16 h at room temperature. Purification by RP-HPLC (40 ‑100% B in 20 min, Method B, 5 mL/min) afforded pure product **S‑7**. MeCN was removed *in vacuo*, residual solvents (H_2_O) were reduced but not completely removed for the next step.

RP-HPLC (40 ‑ 100% B in 15 min, Method A, 1 mL/min): t*_R_*= 28.7 min; *k*= 18. Calculated monoisotopic mass (C_100_H_147_FN_14_O_23_): 1931.07, found: *m/z* = 967.0 [M(**S‑7**)+2H]^2+^, 1932.2 [M(**S‑7**)+H]^+^, 1954.2 [M(**S‑7**)+Na]^+^, 1970.4 [M(**S‑7**)+K]^+^.

*H‑L‑His-Aib‑L‑Glu-Gly‑L‑Thr‑L‑(α-Me)Phe(2-F)‑L‑Thr‑L‑Ser‑L‑Asp‑L‑(2’-Et, 4’-OMe)BIP-L‑homoPhe‑NH_2_ (****1****)*

Peptide **S‑7** in residual H_2_O was dissolved in 500 µL TFA/TIPS/H_2_O (95/2.5/2.5) and stirred for 1 h at room temperature. TFA was removed under a stream of nitrogen, the crude product was frozen (-80 °C) and lyophilized. Purification by preparative RP-HPLC (40 ‑ 55% B in 20 min, Method B, 5 mL/min) afforded 2.05 mg (16.1% referred to **18**) of pure product **1***2TFA as colorless powder.

RP-HPLC (20 ‑ 80% B in 15 min, Method A, 1 mL/min): t*_R_*= 11.6 min; *k*= 6.7. Calculated monoisotopic mass (C_70_H_91_FN_14_O_19_): 1450.66, found: *m/z* = 726.7 [M(**1**)+2H]^2+^, 1452.1 [M(**1**)+H]^+^.

- 1. HPLC chromatograms and mass spectra
     1. Peptide 1

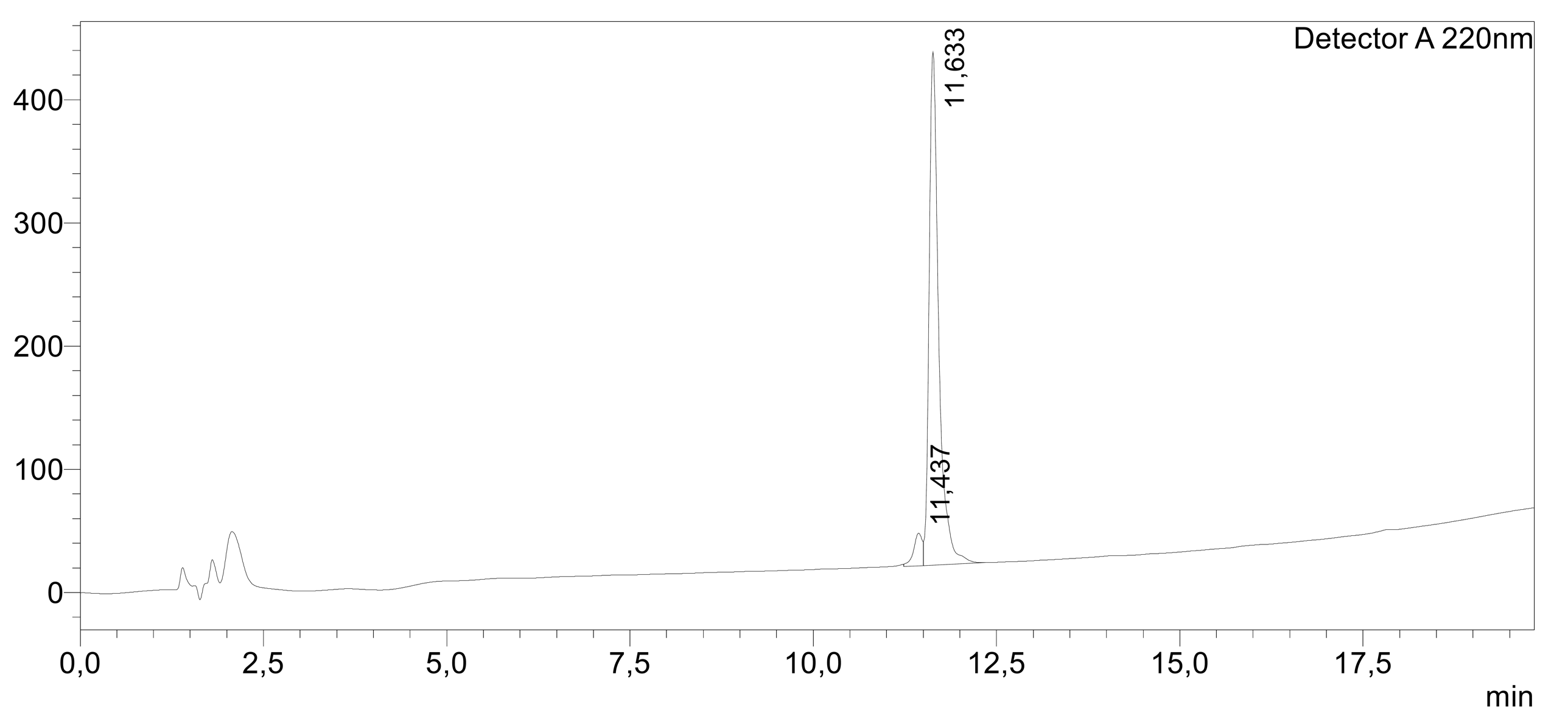


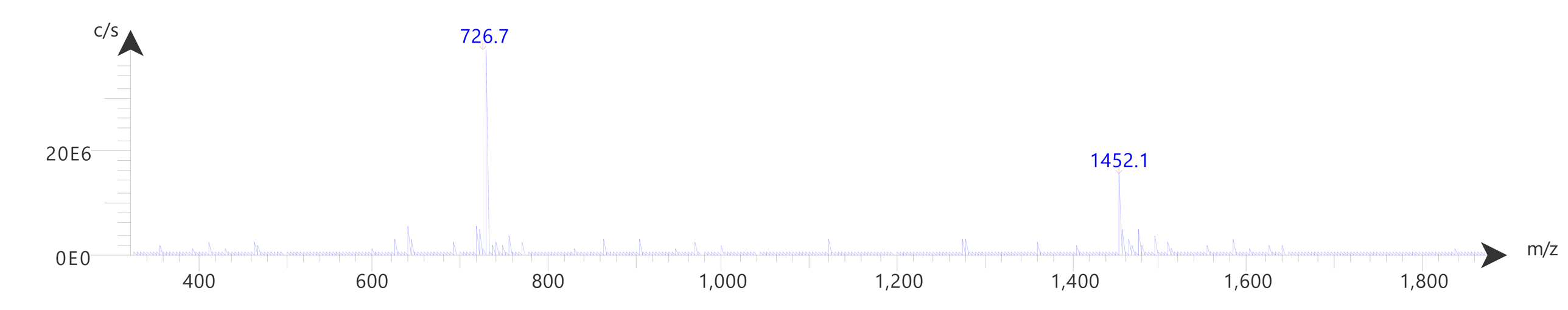


***Figure 1***: HPLC chromatogram of lead peptide **1** (above) and associated mass spectrum (below). Gradient: 20 ‑ 80% B in 15 min, Method A, 1 mL/min.

- - 1. Peptide 2


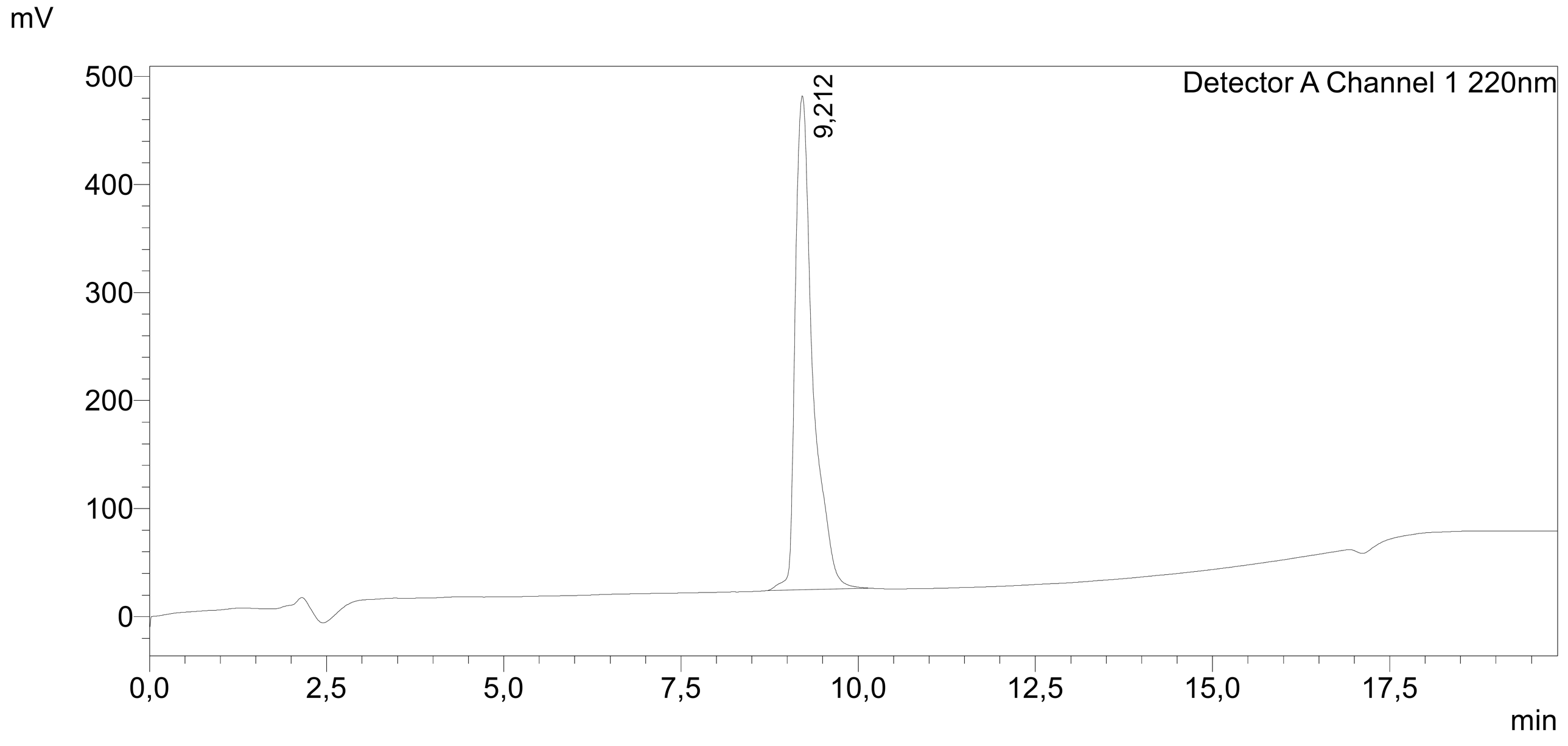

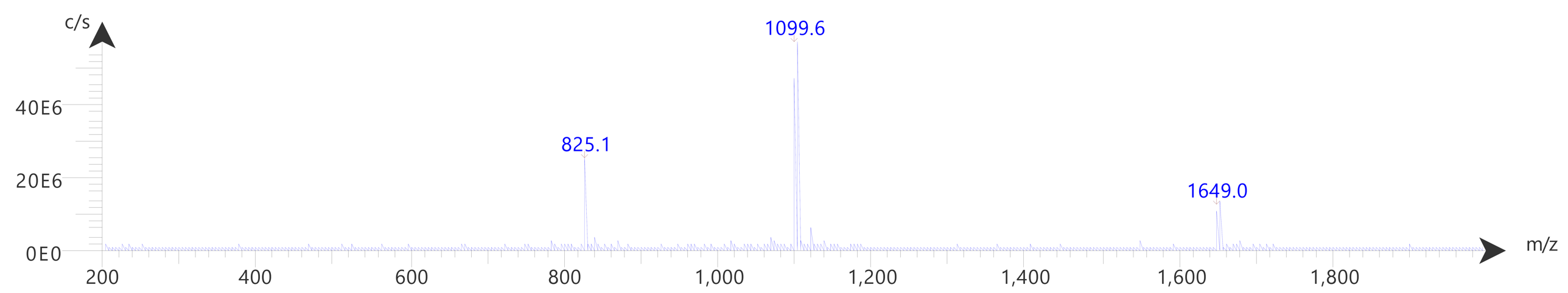
***Figure 2***: HPLC chromatogram of GLP-1 (**2**) (above) and associated mass spectrum (below). Gradient: 10 ‑ 90% B in 15 min, Method A, 1 mL/min.

- - 1. Reference radioligand [^125^I]Tyr(3‑I)^40^‑3

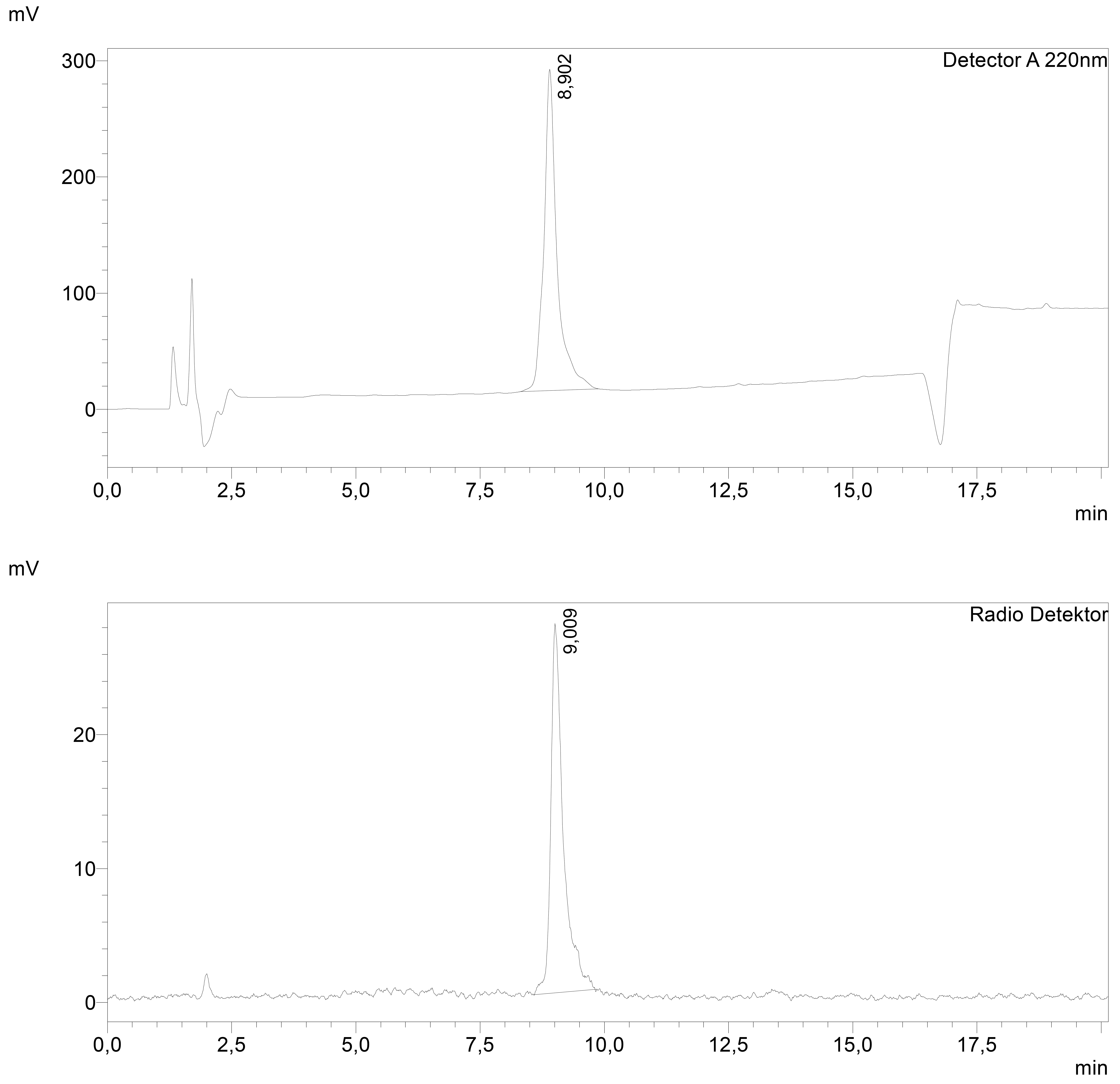


***Figure 3***: Radio-RP-HPLC chromatogram of reference radioligand [^125^I]Tyr(3‑I)^40^‑**3** at the day of radioiodination (below), with co‑injected cold-standard Tyr(3‑I)^40^‑**3**, visible in the UV/Vis channel (above). Gradient: 20 ‑ 70% B in 15 min, Method A, 1 mL/min.

***Table 1***: Stability study of reference radioligand [^125^I]Tyr(3‑I)^40^]‑**3**, (n = 1).

| Days after radioiodination | RCP of [^125^I]Tyr(3‑I)^40^]‑3^a^ |
| --- | --- |
| 0 | ≥ 95% (n = 4) |
| 4 | 95% |
| 9 | 95% |
| 16 | 92% |
| 20 | 82% |
| 24 | 82% |
| 29 | 78% |
| 36 | 78% |

^a^n = 1, unless otherwise stated.

- - 1. Peptide 4

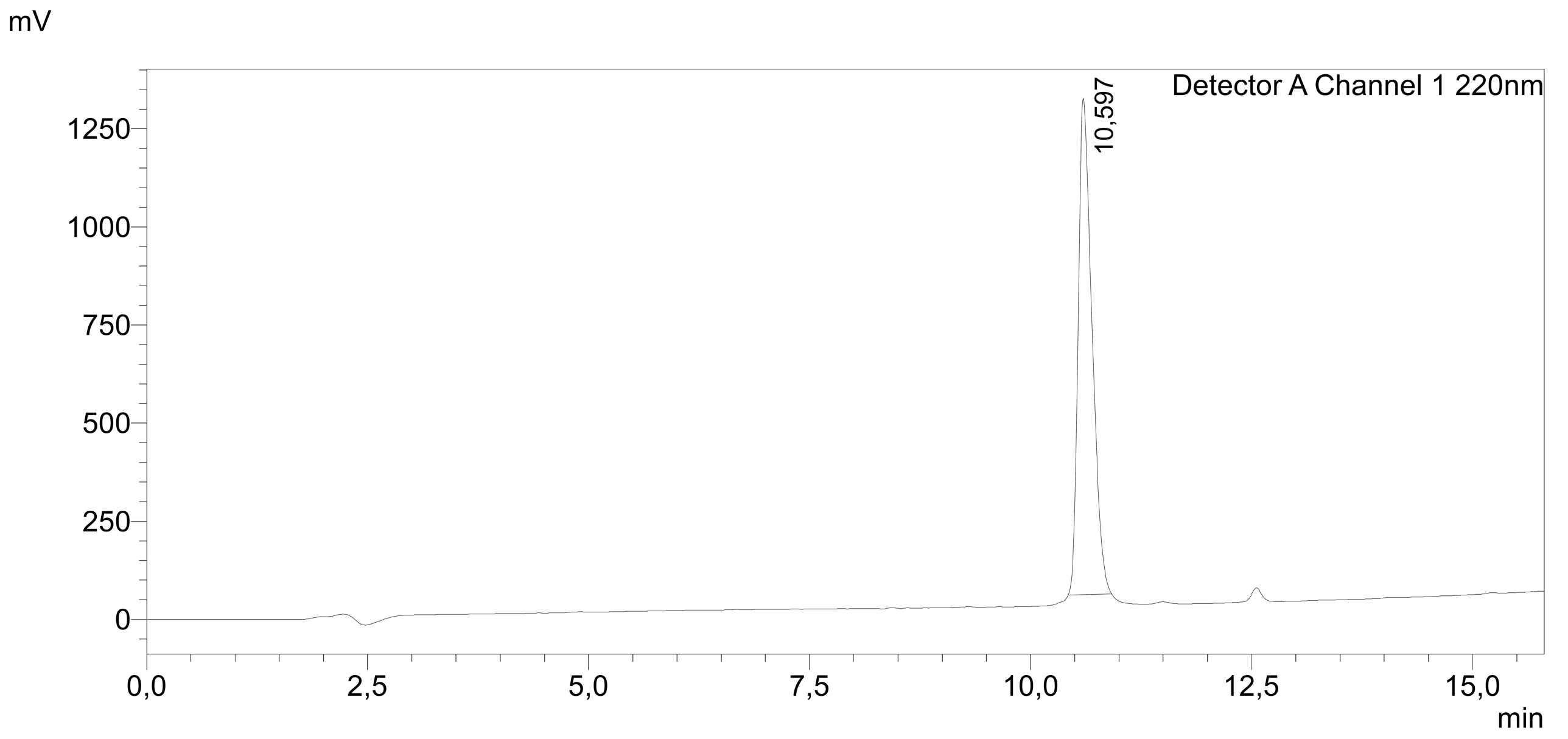


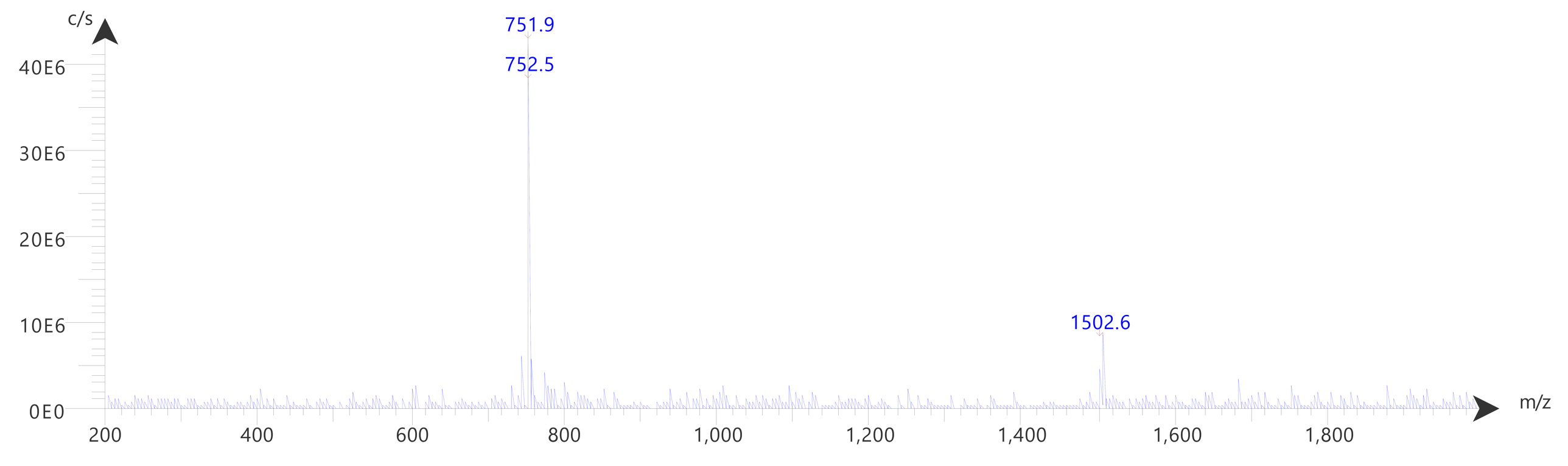


***Figure 4***: HPLC chromatogram of SiFA-tagged undecapeptide **4** (above) and associated mass spectrum (below). Gradient: 10 ‑ 90% B in 15 min, Method A, 1 mL/min.

- - 1. Peptide 5

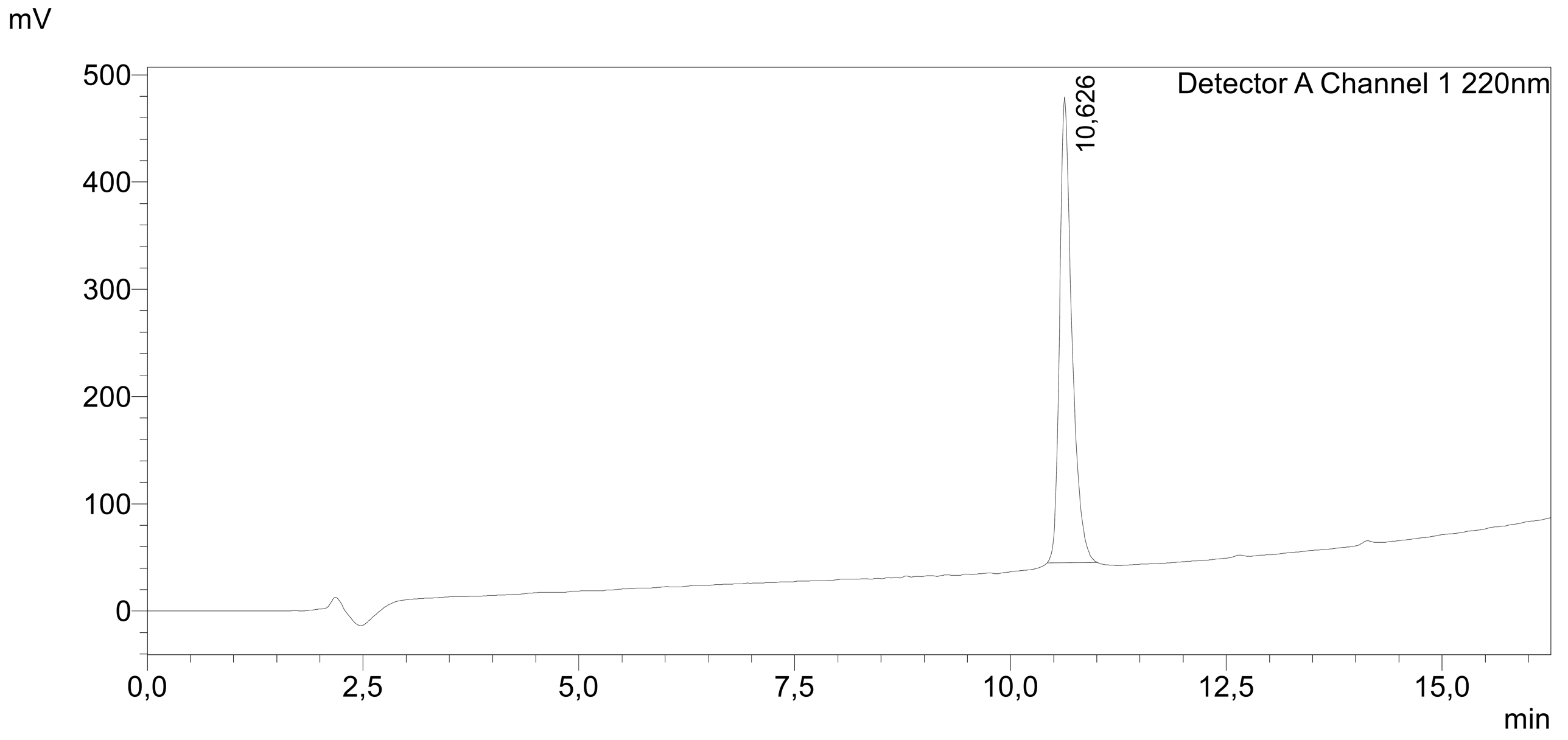


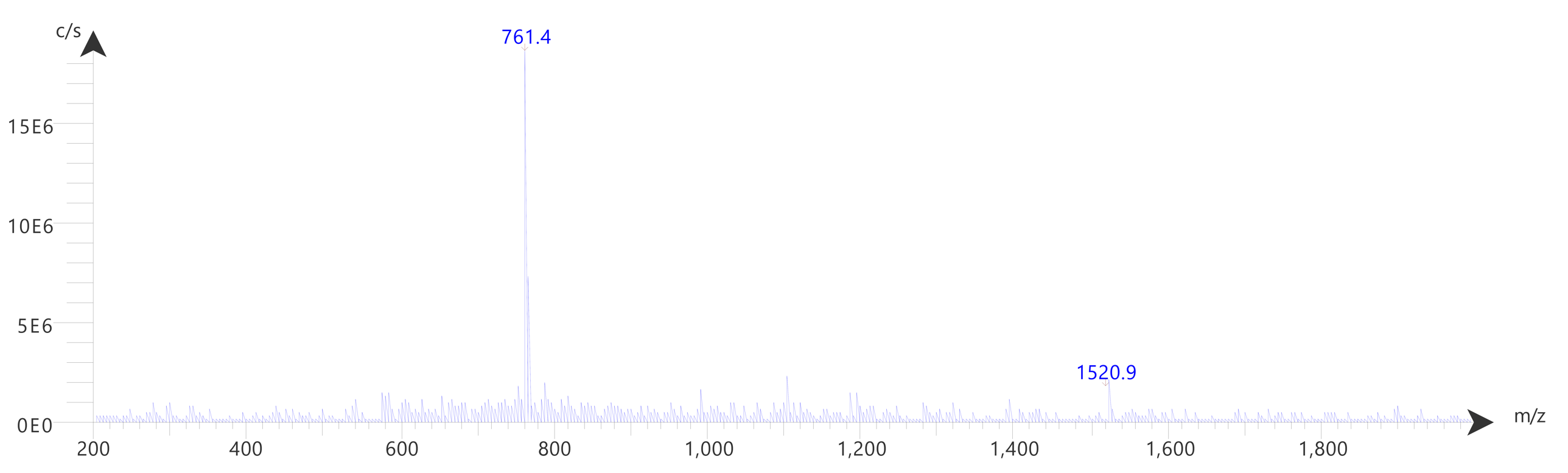


***Figure 5***: HPLC chromatogram of SiFA-tagged undecapeptide **5** (above) and associated mass spectrum (below). Gradient: 10 ‑ 90% B in 15 min, Method A, 1 mL/min.

- - 1. Peptide 6

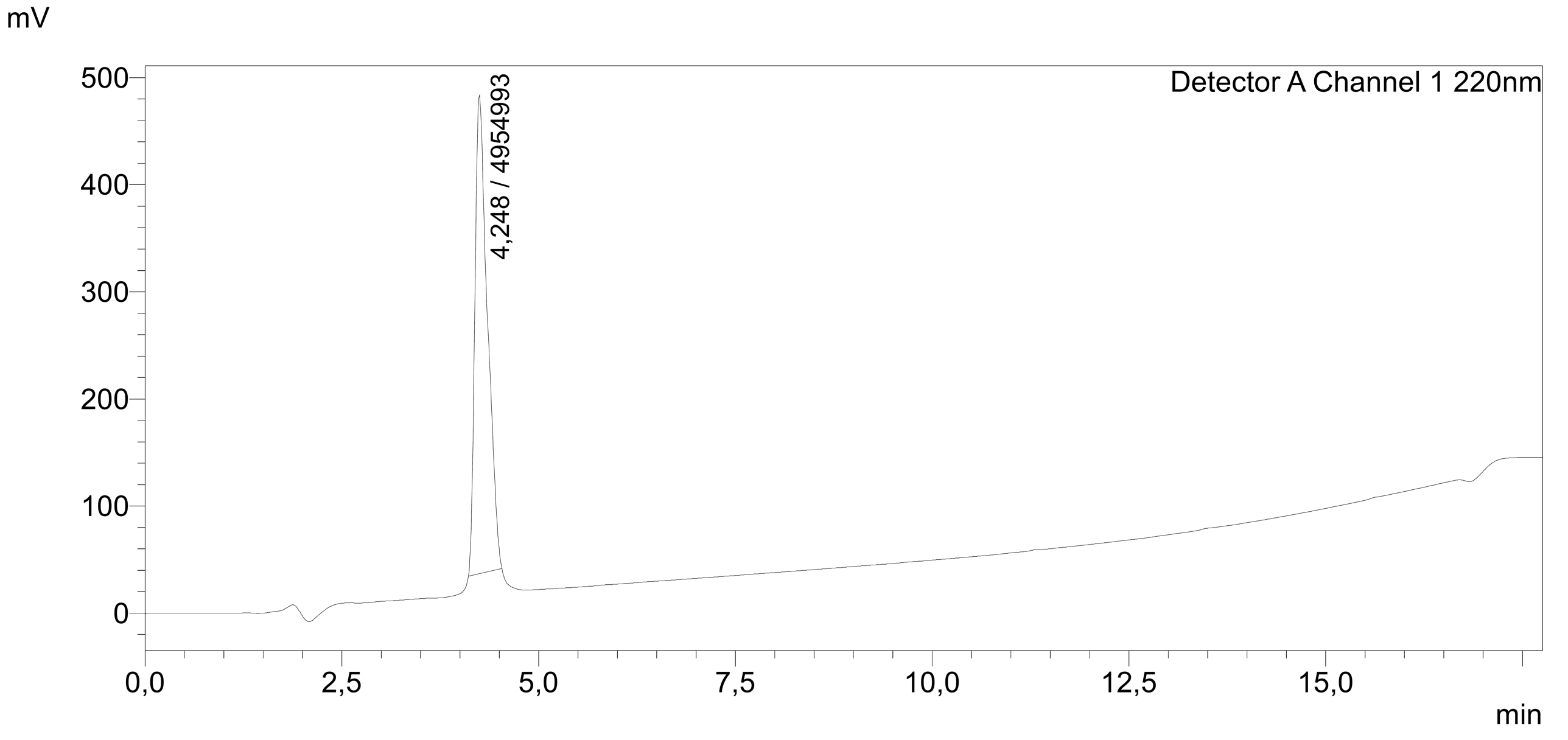


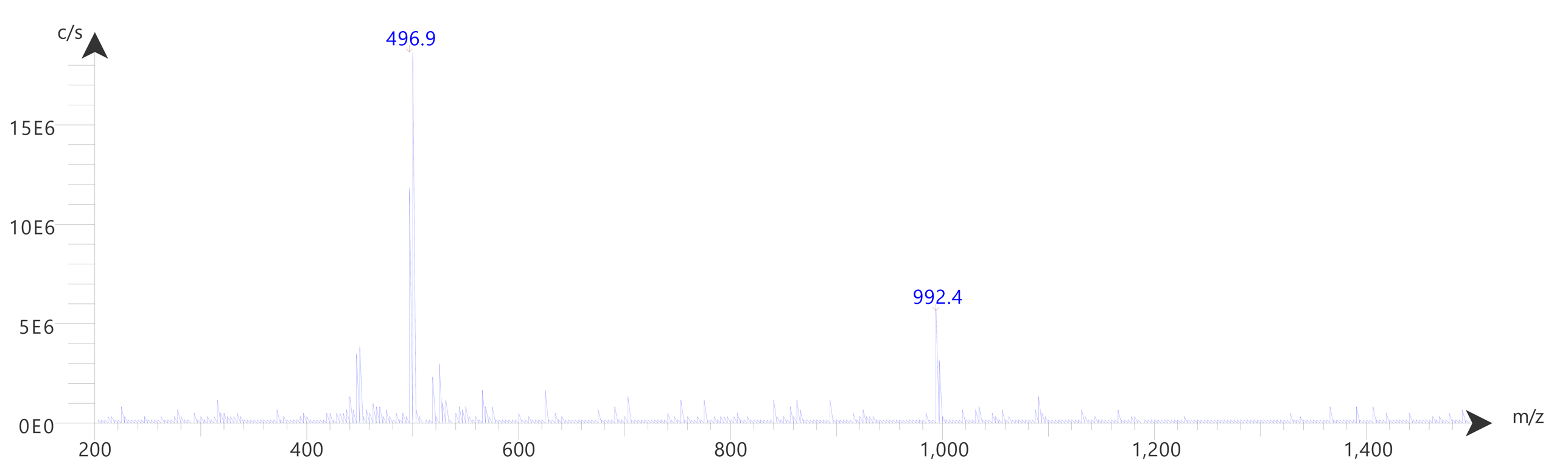


***Figure 6***: HPLC chromatogram of peptide **6** (above) and associated mass spectrum (below). Gradient: 10 ‑ 90% B in 15 min, Method A, 1 mL/min.

- - 1. Peptide 7

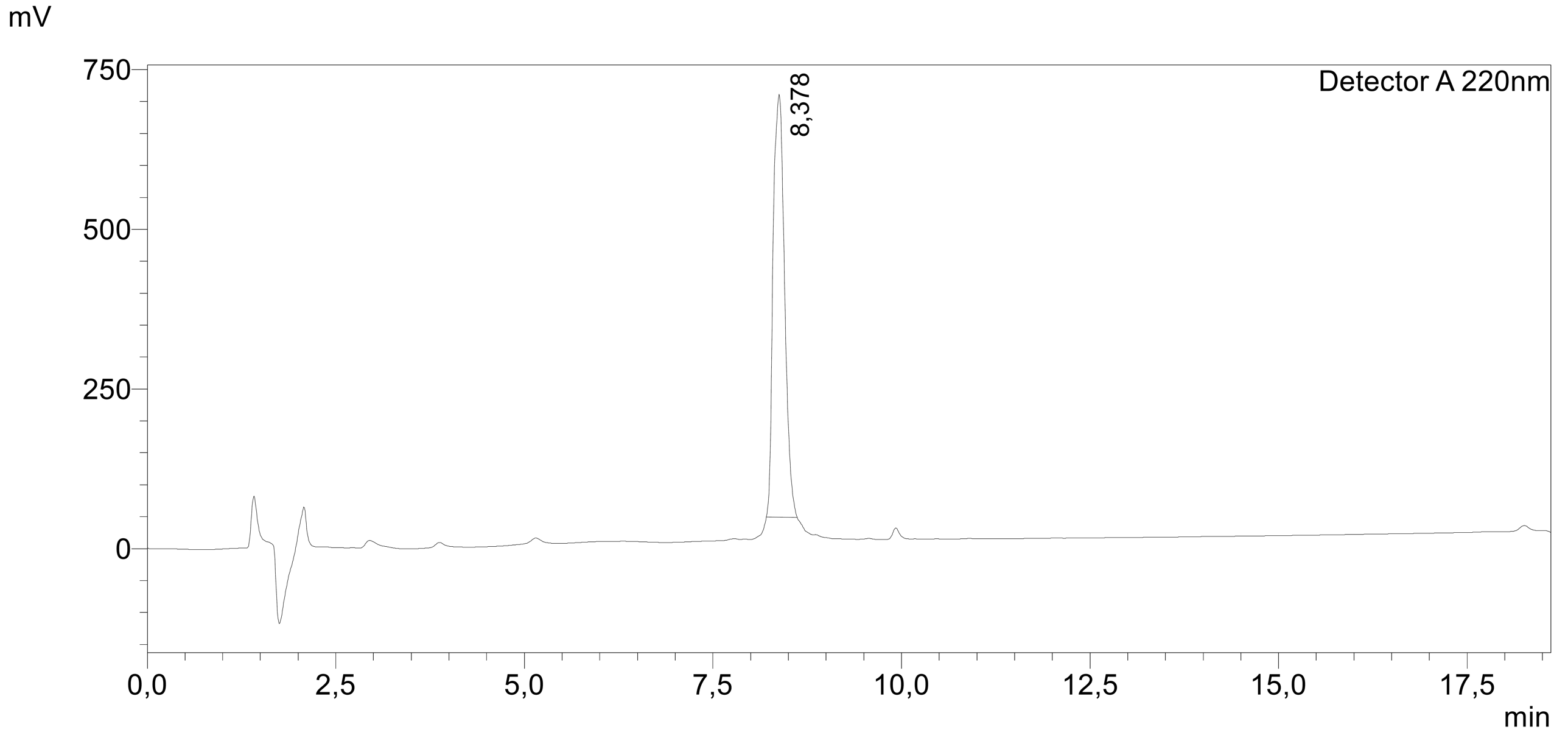


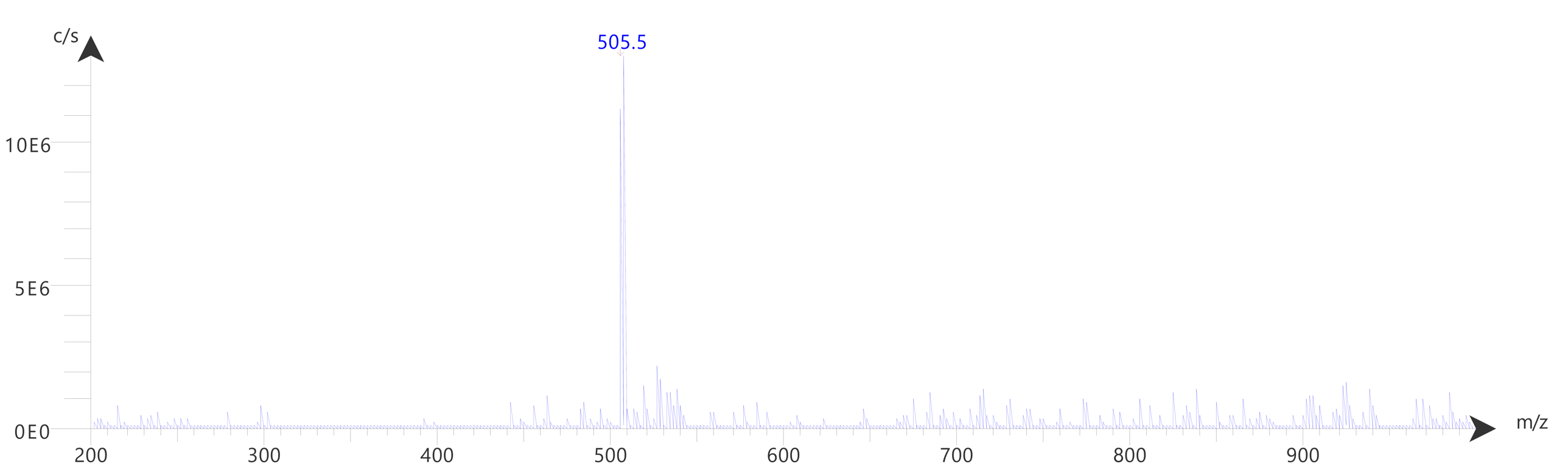


***Figure 7***: HPLC chromatogram of peptide **7** (above) and associated mass spectrum (below). Gradient: 10 ‑ 60% B in 15 min, Method A, 1 mL/min.

- - 1. Peptide 8

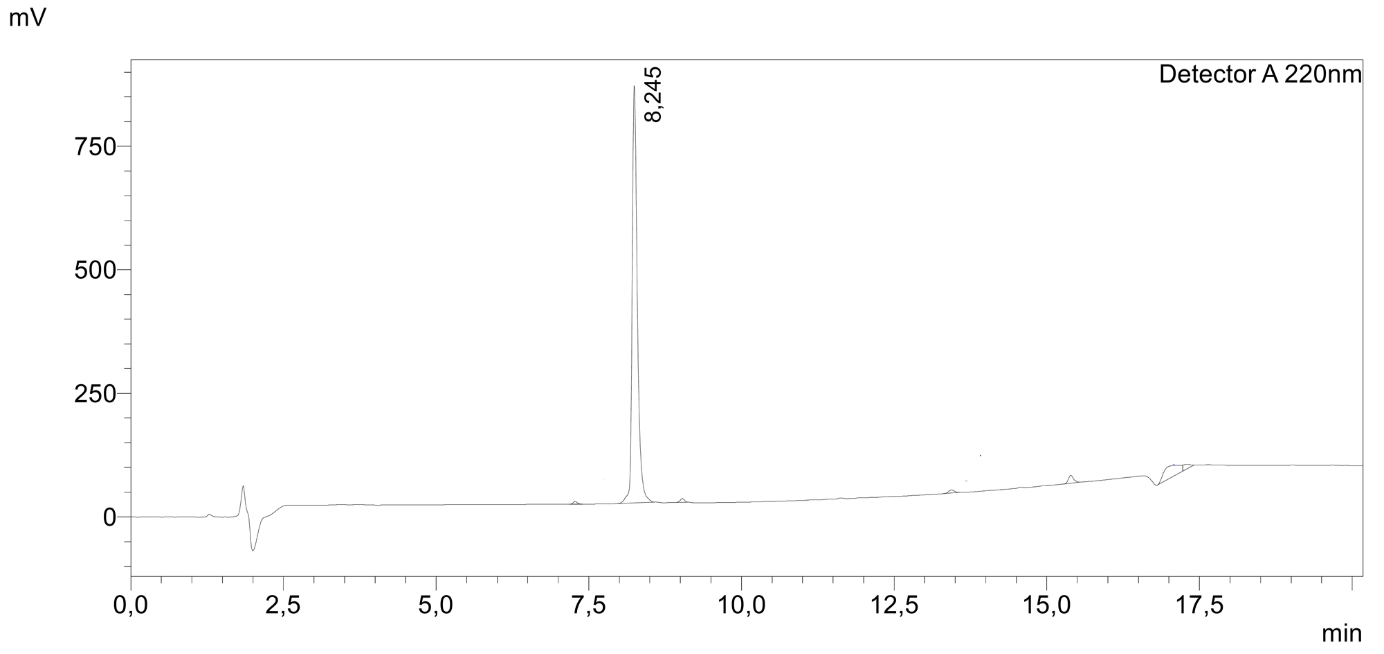

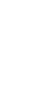

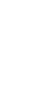


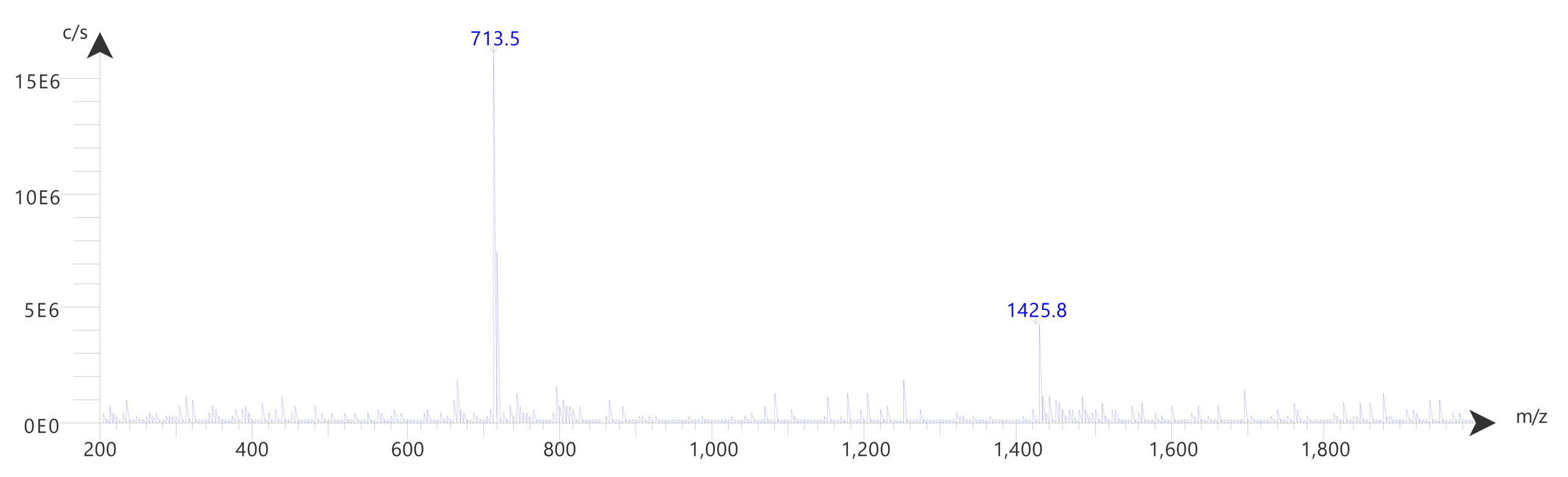


***Figure 8***: HPLC chromatogram of peptide **8** (above) and associated mass spectrum (below). Gradient: 10 ‑ 90% B in 15 min, Method A, 1 mL/min.

- - 1. Peptide 9

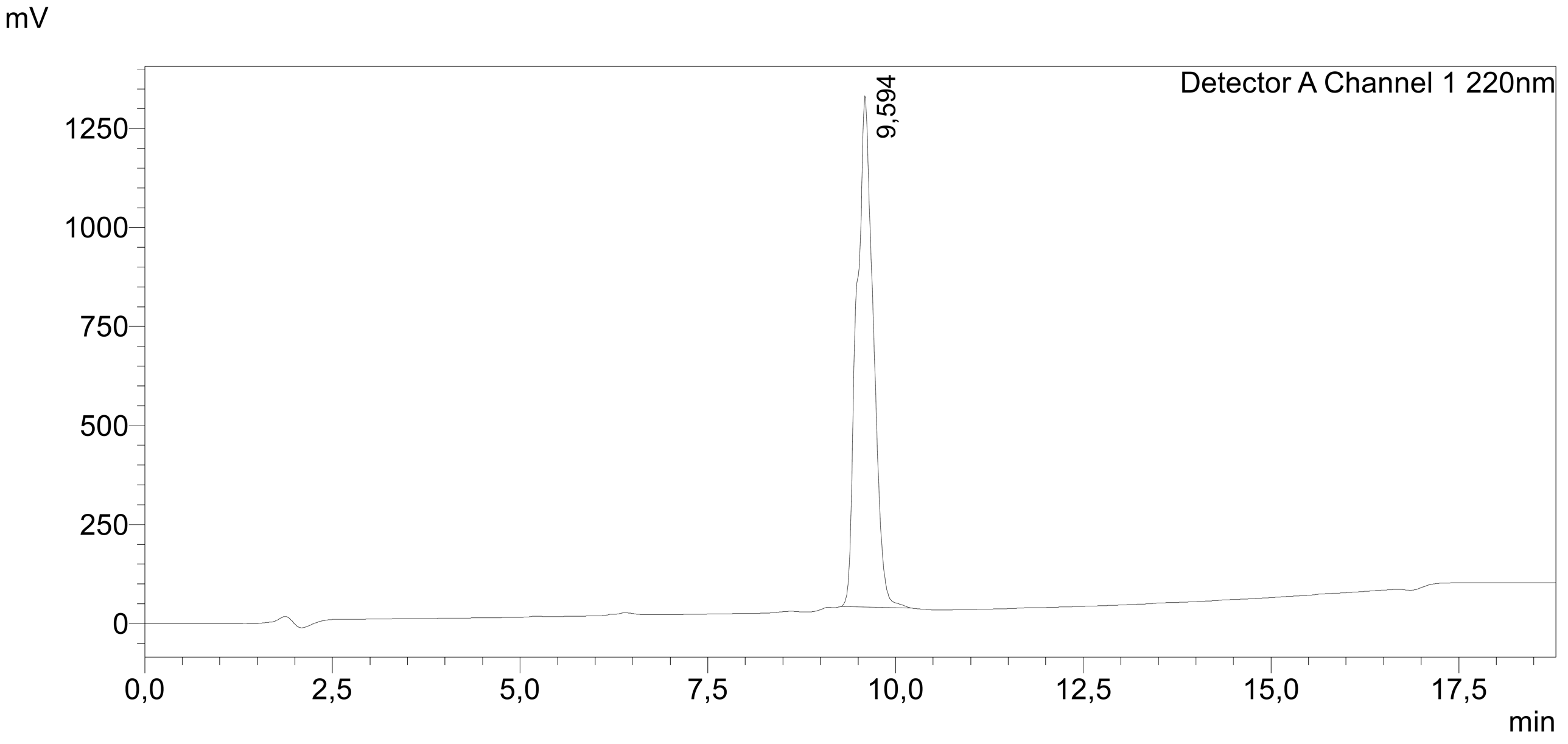


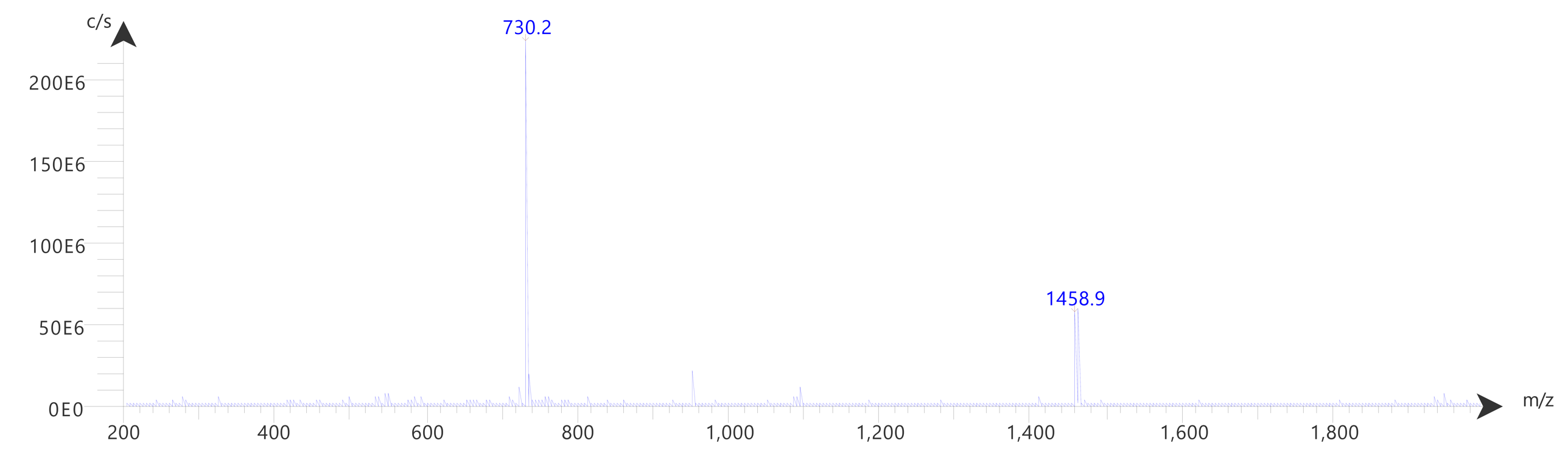


***Figure 9***: HPLC chromatogram of peptide **9** (above) and associated mass spectrum (below). Gradient: 10 ‑ 90% B in 15 min, Method A, 1 mL/min.

- - 1. Peptide 10

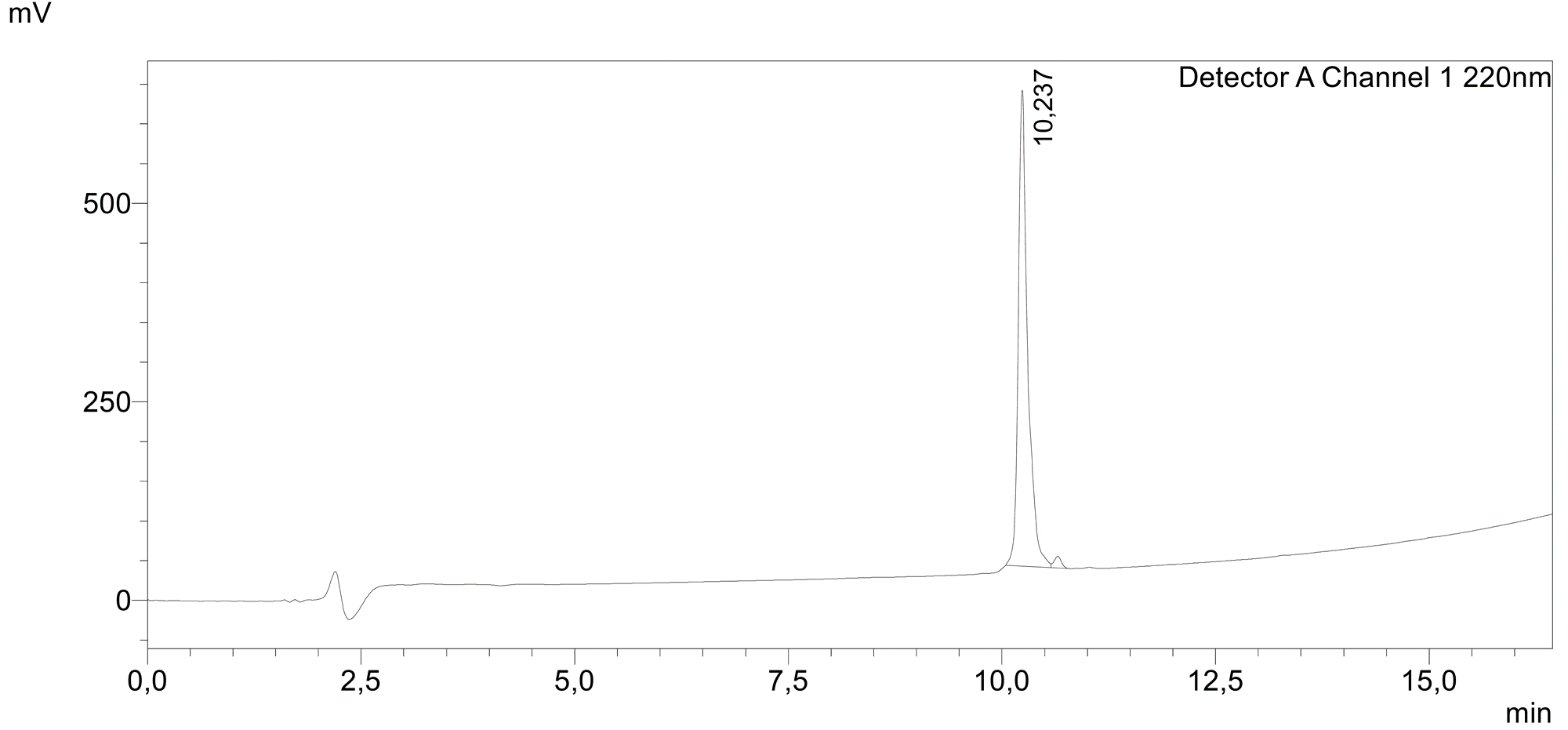


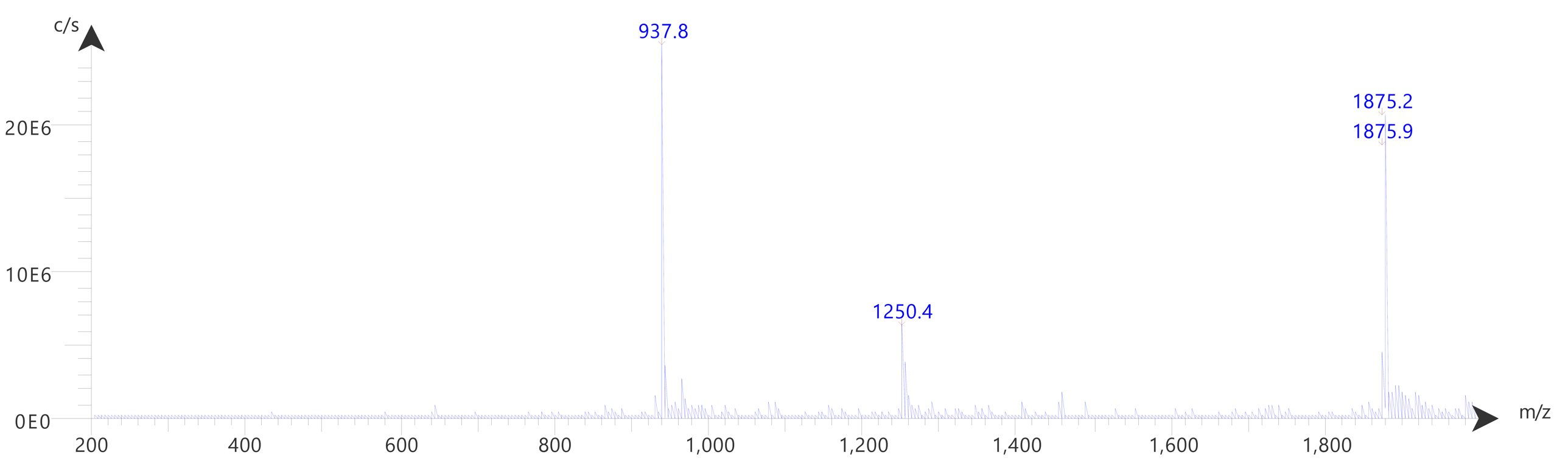


***Figure 10***: HPLC chromatogram of peptide **10** (above) and associated mass spectrum (below). Gradient: 10 ‑ 90% B in 15 min, Method A, 1 mL/min.

- - 1. Peptide 11

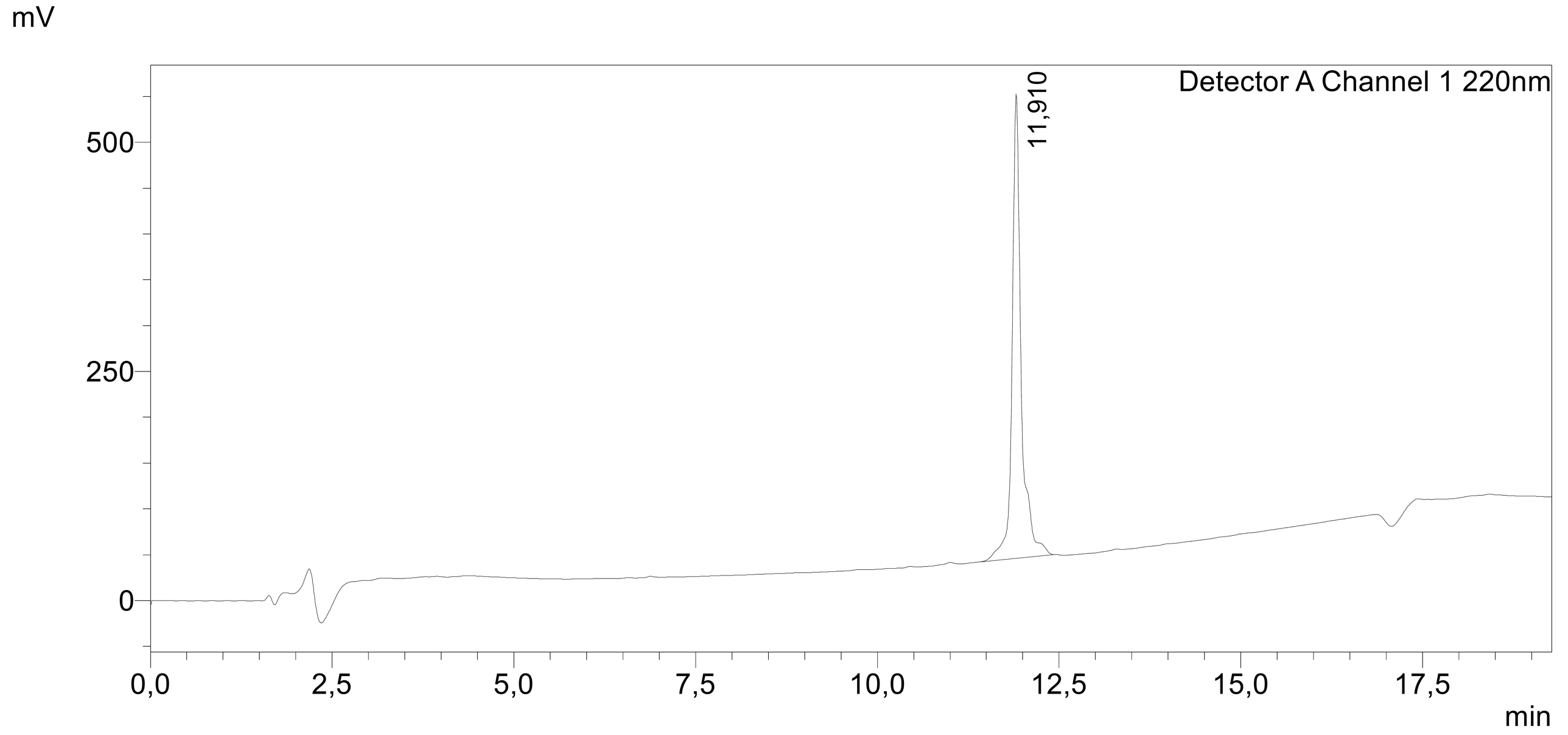


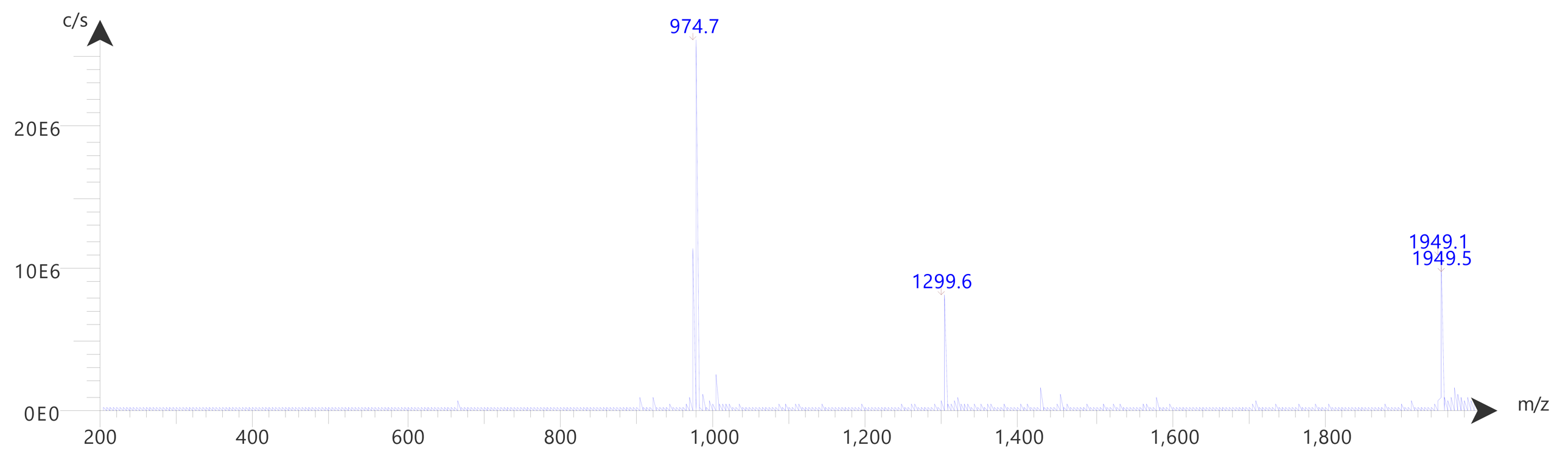


***Figure 11***: HPLC chromatogram of peptide **11** (above) and associated mass spectrum (below). Gradient: 10 ‑ 90% B in 15 min, Method A, 1 mL/min.

- - 1. Peptide 12

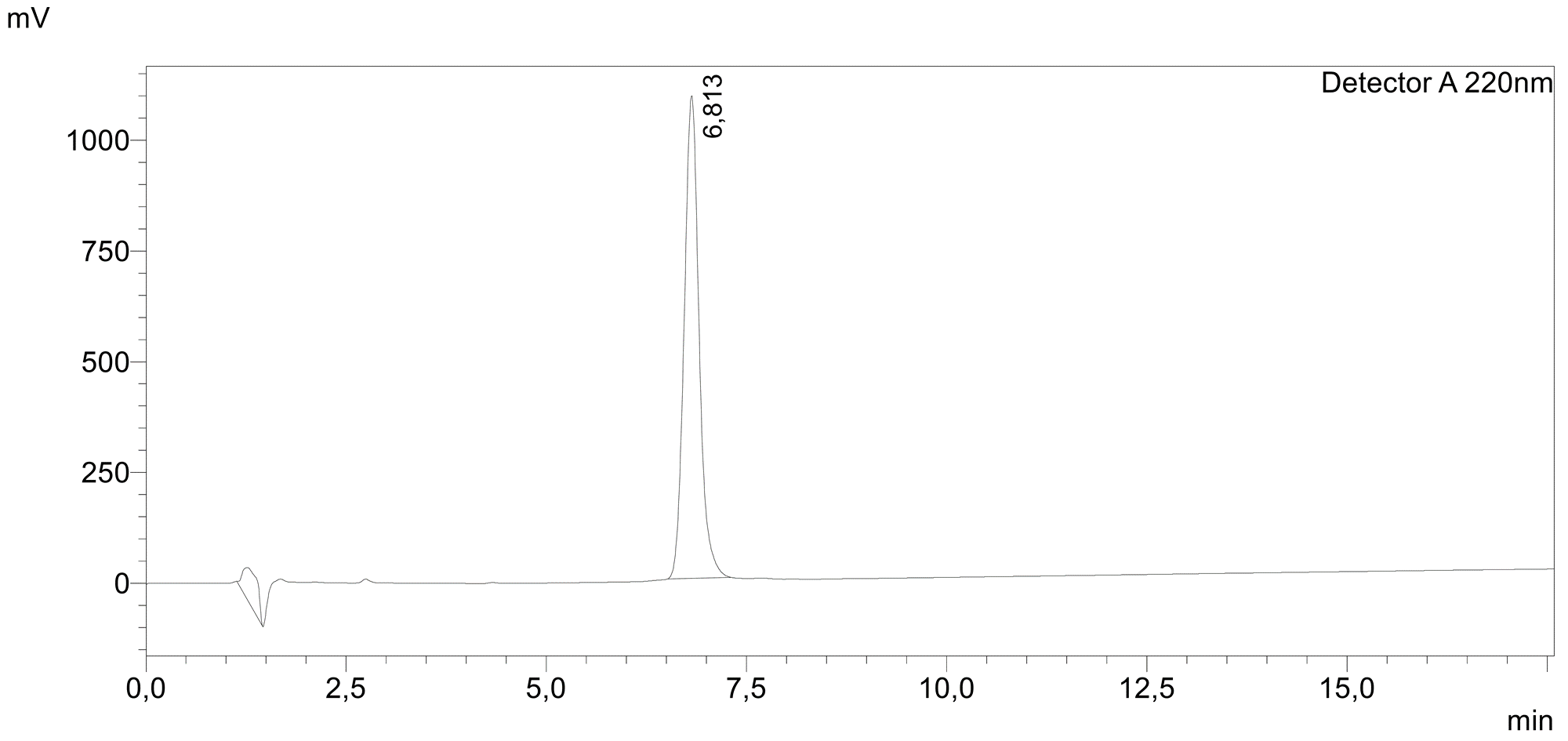


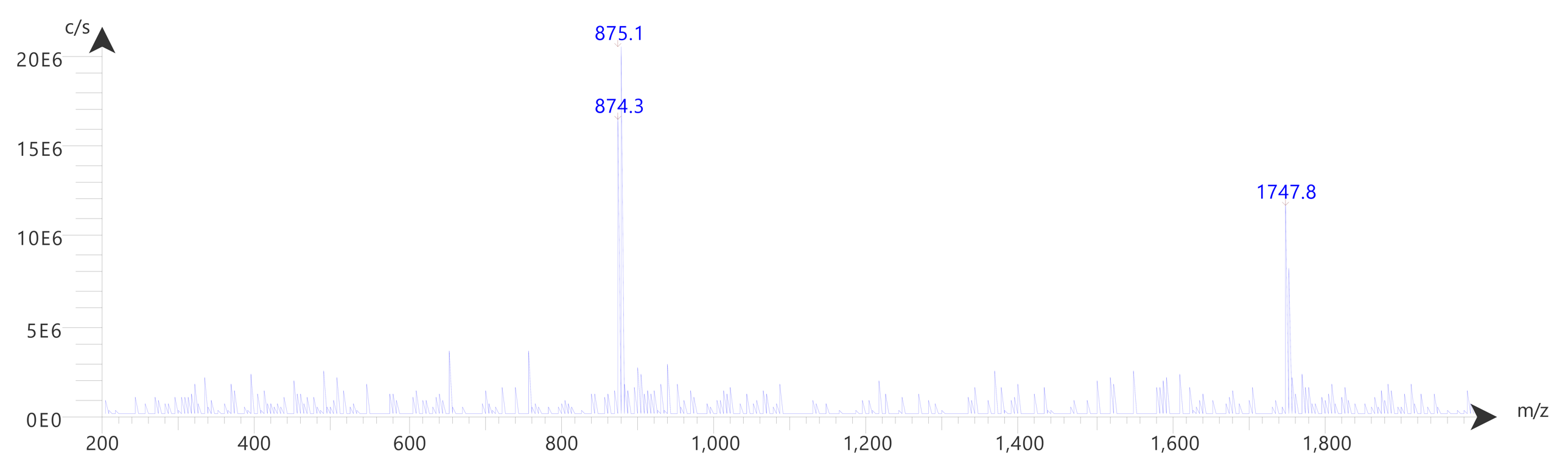


***Figure 12***: HPLC chromatogram of peptide **12** (above) and associated mass spectrum (below). Gradient: 80 ‑ 100% B in 15 min, Method B, 1 mL/min.

- - 1. Peptide 13

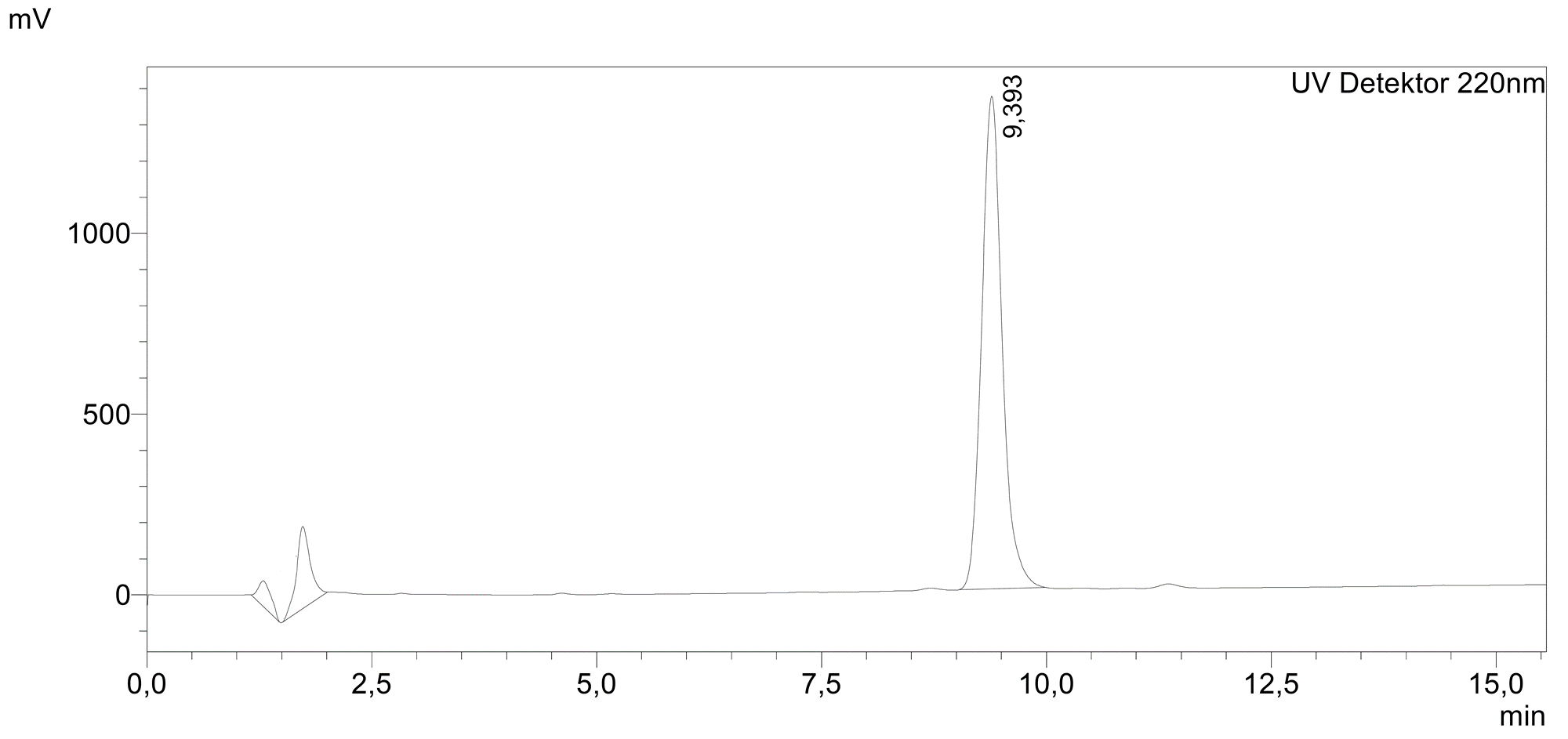


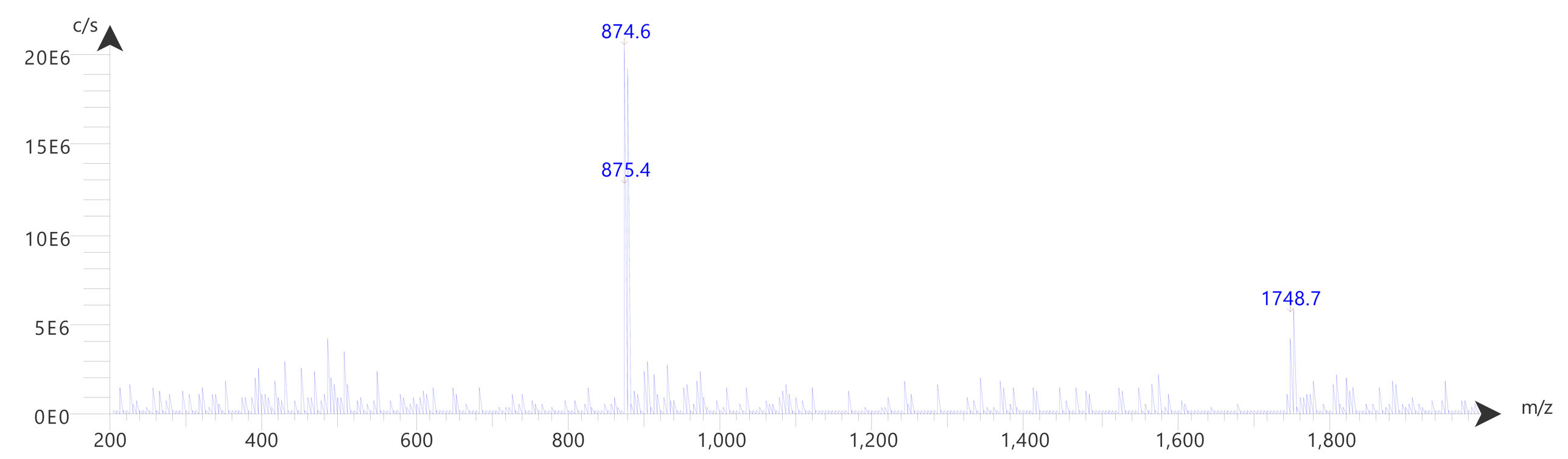


***Figure 13***: HPLC chromatogram of peptide **13** (above) and associated mass spectrum (below). Gradient: 80 ‑ 100% B in 15 min, Method B, 1 mL/min.

- - 1. Peptide 14

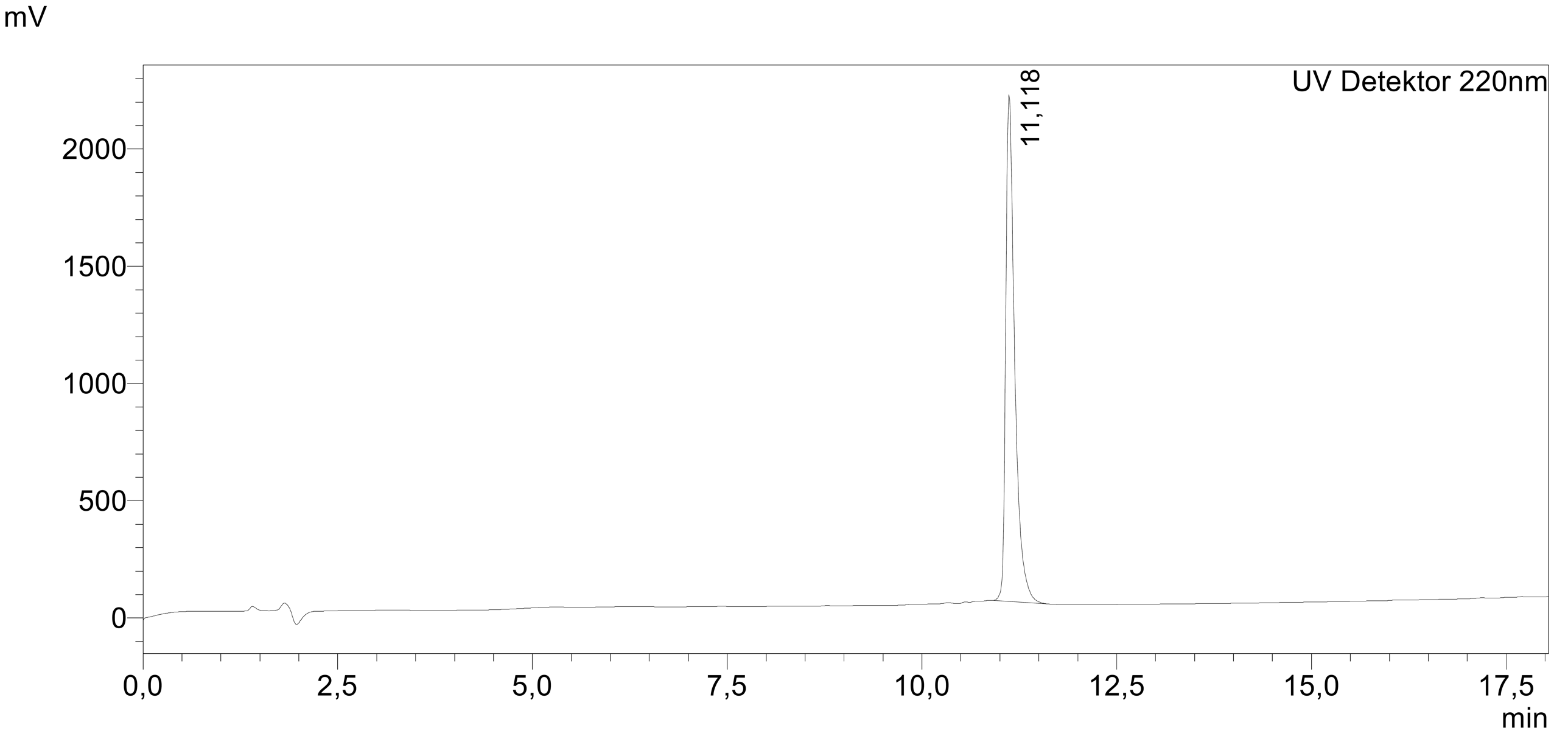


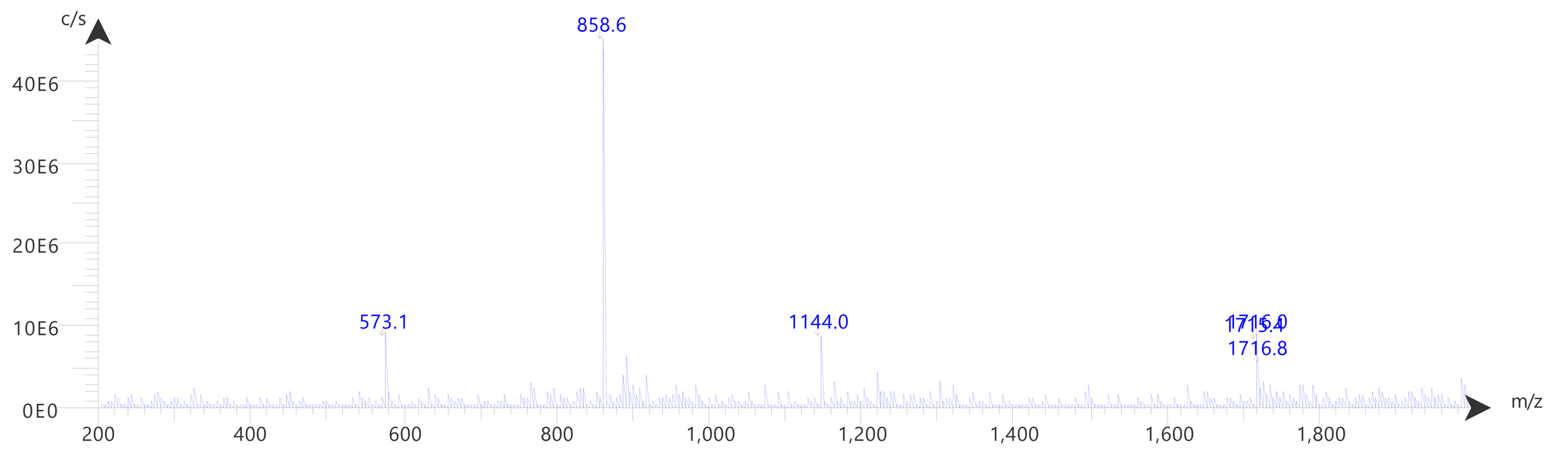


***Figure 14***: HPLC chromatogram of peptide **14** (above) and associated mass spectrum (below). Gradient: 10 ‑ 90% B in 15 min, Method B, 1 mL/min.

- 1. *In vitro* experiments
     1. Cell culture

HEK293-hGLP-1R cells (HEK293 cells stably transfected with the human GLP‑1 receptor)^(3)^ were kindly provided by Prof. Dr. Timothy J. Kieffer (University of British Columbia, Vancouver, Canada). Cells were cultivated in high glucose DMEM (25.0 mM D-glucose, 3.97 mM GlutaMAX; REF-number: 61965-026; Fisher Scientific GmbH, Schwerte, Germany) supplemented with 10% fetal bovine serum and 1 mM sodium pyruvate. Medium for stably transfected HEK293‑hGLP-1R cells was constantly supplemented with 1 mg/mL Geneticin (G‑418 Biochrom, Merck KgaA, Darmstadt, Germany) as selection antibiotic. The cell line was kept at 37 °C in a humidified 5% CO_2_ atmosphere. One day (24 ± 2 h) prior to all *in vitro* experiments, the cultivated cells were harvested using a mixture of trypsin/ethylenediaminetetraacetic acid (0.05%/0.02%) in phosphate-buffered saline (PBS) (Merck KgaA, Darmstadt, Germany) and centrifuged at 1300 rpm (ca. 190 × g) for 3 min at room temperature (Heraeus Megafuge 16, Thermo Fisher Scientific, Darmstadt, Germany). After centrifugation, the supernatant was disposed and the cell pellet was resuspended in culture medium. Cells were counted with a Neubauer hemocytometer (Paul Marienfeld GmbH & Co. KG, Lauda‑Königshofen, Germany) and seeded in 24-well plates (Greiner Bio‑One, Kremsmünster, Austria). IC_50_ values were determined by transferring 1.50 × 10^5^ cells/mL per well into 24‑well plates (Greiner Bio‑One, Kremsmünster, Austria).

- - 1. Affinity determinations (IC_50_)

The culture medium was removed and the cells were washed with 500 µL of Hank’s balanced salt solution (HBSS) (Merck KGaA, Darmstadt, Germany), containing 1% bovine serum albumin (BSA) (Merck KGaA, Darmstadt, Germany). Afterwards, 200 µL of HBSS (1% BSA) were added to each well and equilibrated on ice (4 °C) for 15 min. 25 µL/well of either HBSS (1% BSA) (= control) or of solutions, containing the respective unlabeled ligand in increasing concentrations (10^-10^- 10^-4^ M in HBSS) were added, followed by the addition of 25 µL of [Nle^14^, [^125^I]Tyr(3-I)^40^]exendin-4 in HBSS (1% BSA) to each well. Experiments were carried out in triplicates for each concentration. The final concentrations of unlabeled ligand ranged from 10^-11^- 10^-5^ M and the final radioligand concentration was 0.41 nM in all binding assays. The cells were incubated for two hours at 4 °C. Incubation was terminated by removal of the incubation medium. The cells were washed with 250 µL of HBSS (1% BSA) and the wash medium was combined with the respective supernatant. This fraction represents the amount of free radioligand. The cells were lysed by addition of 250 µL of 1 M aqueous NaOH. After 20 min, the lysate of each well was transferred to the respective vial as well as 250 µL of 1 M NaOH used for rinsing the well. Quantification of the amount of free and bound activity was performed in a γ-counter. The corresponding IC_50_ values were calculated using the GraphPad PRISM7 software.

1. ABBREVIATIONS

2-CT 2-chlorotrityl

Ac acetyl

AcOH acetic acid

Aib α-aminoisobutyric acid

BIP 4, 4’‑biphenylalanine

Boc *tert*-butyloxycarbonyl

BSA bovine serum albumin

CT computed tomography

Dap 2,3-diaminopropionic acid

DCM dichloromethane

Dde *N*-1-(4,4-dimethyl-2,6-dioxocyclohex-1-ylidene)ethylamine

DIC *N,N′*-diisopropylcarbodiimide

DIPEA *N*,*N*-diisopropylethylamine

DMA dimethyl acetamide

DMEM Dulbecco's Modified Eagle's Medium

DMF dimethylformamide

DMSO dimethyl sulfoxide

EC_50_ half maximal effective concentration

eq. equivalent(s)

*er* enantiomeric ratio

Et_2_O diethyl ether

Fmoc fluorenylmethoxycarbonyl

GLP-1 glucagon-like peptide 1

GLP-1R glucagon-like peptide 1 receptor

HATU *O*-(7-Azabenzotriazol-1-yl)-*N,N,N',N'*-tetramethyluronium hexafluorophosphat

HBSS Hank’s buffered salt solution

HEK293 human embryonic kidney 293 cells

HFIP 1,1,1,3,3,3-hexafluoro-2-propanol

hGLP-1R human glucagon-like peptide 1 receptor

HOAt 1-hydroxy-7-azabenzotriazole

HOBt 1-hydroxybenzotriazole

IC_50_ half maximal inhibitory concentration

MeCN acetonitrile

MeOH methanol

NMP *N*-methyl-2-pyrrolidone

O2Oc 8-amino-3,6-dioxaoctanoic acid

PBS phosphate-buffered saline

PET positron emission tomography

PPh_3_ triphenylphosphane

PyBOP benzotriazol-1-yl-oxytripyrrolidinophosphonium hexafluorophosphate

RACM Rink amide ChemMatrix^®^

RP‑HPLC reversed-phase high-performance liquid chromatography

r.t. room temperature

SiFA-BA silicon-based fluoride acceptor-benzoic acid

SPECT single-photon emission computed tomography

TBTU 2-(1*H*-benzotriazole-1-yl)-1,1,3,3-tetramethylaminium tetrafluoroborate

*t*Bu *tert*-butyl

TFA trifluoroacetic acid

TIPS triisopropylsilane

TLC thin layer chromatography

t*_R_* retention time (reversed-phase high-performance liquid chromatography)

1. REFERENCES

1. Haque TS, Martinez RL, Lee VG, Riexinger DG, Lei M, Feng M, et al. Exploration of structure–activity relationships at the two C-terminal residues of potent 11mer Glucagon-Like Peptide-1 receptor agonist peptides via parallel synthesis. Peptides. 2010;31(7):1353-60.

2. Haque TS, Lee VG, Riexinger D, Lei M, Malmstrom S, Xin L, et al. Identification of potent 11mer Glucagon-Like Peptide-1 Receptor agonist peptides with novel C-terminal amino acids: Homohomophenylalanine analogs. Peptides. 2010;31(5):950-5.

3. Gromada J, Rorsman P, Dissing S, Wulff BS. Stimulation of cloned human glucagon-like peptide 1 receptor expressed in HEK 293 cells induces cAMP-dependent activation of calcium-induced calcium release. FEBS Letters. 1995;373(2):182-6.
